# Evolution of drug‐tolerant nematode populations in response to density reduction

**DOI:** 10.1111/eva.12376

**Published:** 2016-03-29

**Authors:** Alan Reynolds, Jan Lindström, Paul C. D. Johnson, Barbara K. Mable

**Affiliations:** ^1^ Institute of Biodiversity, Animal Health and Comparative MedicineCollege of Medical, Veterinary and Life SciencesUniversity of GlasgowGlasgowUK

**Keywords:** *Caenorhabditis remanei*, drug resistance, drug tolerance, experimental evolution, pesticide resistance, population density, rapid evolution, selection experiment

## Abstract

Resistance to xenobiotics remains a pressing issue in parasite treatment and global agriculture. Multiple factors may affect the evolution of resistance, including interactions between life‐history traits and the strength of selection imposed by different drug doses. We experimentally created replicate selection lines of free‐living *Caenorhabditis remanei* exposed to Ivermectin at high and low doses to assess whether survivorship of lines selected in drug‐treated environments increased, and if this varied with dose. Additionally, we maintained lines where mortality was imposed randomly to control for differences in density between drug treatments and to distinguish between the evolutionary consequences of drug‐treatment versus ecological processes due to changes in density‐dependent feedback. After 10 generations, we exposed all of the selected lines to high‐dose, low‐dose and drug‐free environments to evaluate evolutionary changes in survivorship as well as any costs to adaptation. Both adult and juvenile survival were measured to explore relationships between life‐history stage, selection regime and survival. Intriguingly, both drug‐selected and random‐mortality lines showed an increase in survivorship when challenged with Ivermectin; the magnitude of this increase varied with the intensity of selection and life‐history stage. Our results suggest that interactions between density‐dependent processes and life history may mediate evolved changes in susceptibility to control measures.

## Introduction

Pesticide and drug treatments are designed to suppress populations of parasites, pests and disease vectors. This makes them strong selective factors; as a result, adaptation consistently occurs in natural populations exposed to xenobiotics (Jackson 1993; Carriere et al. 1994; Wolstenholme et al. 2004; Sparks et al. 2012). Resistance can evolve quickly (Lopes et al. 2008; Brausch and Smith 2009; Tabashnik et al. 2014), and the development of resistance is becoming an important theme in applied evolutionary biology due to the risk of reduced efficacy of chemical applications to control parasite and pest species (Palumbi and Mu 2001; REX Consortium 2010, 2013; Hendry et al. 2011). However, evolutionary strategies which could curtail the rate of resistance evolution have yet to be adopted universally (Greene et al. 2012). Several factors are known to affect the rate at which parasites can evolve resistance, including the type of drug, dosage, timing of application, migration rates between susceptible and resistant populations, the standing frequency of resistance alleles in the population and the specific mechanisms of resistance (Committee on Strategies for the Management of Pesticide Resistant Pest Populations 1986; Barnes et al. 1995; Gilleard and Beech 2007; James et al. 2009; REX Consortium 2013). Low population densities in drug‐treated environments may also have some influence on susceptibility if there are interactions between susceptibility and competition for resources or any other density‐dependent processes. However, it is difficult to tease apart the effects of mortality caused by the drug from those caused by density‐dependence (Gilleard and Beech 2007). In addition, life‐history characteristics and reproductive strategies of parasites and pests could influence the rate at which resistance develops (Galvani and Gupta 1998; Lynch et al. 2008; Kliot and Ghanim 2012). The influence of such factors, and their interactions, on resistance evolution has been considered theoretically but there has been little attempt to show that these factors are of practical significance in the laboratory or field.

Experimentation and monitoring of complicated host–parasite systems is technically difficult, expensive and time‐consuming (Leathwick et al. 2009) and thus resistance evolution is often predicted by simulations. For example, Barnes et al. (1995) used mathematical modeling to investigate the effects of under‐dosing on the evolution of resistance. They suggested that the outcome of under‐dosing in terms of the rate of resistance evolution would depend on the genetic mechanism underlying resistance. An alternative to allow specific testing of factors associated with resistance while maintaining more biological complexity, is to use laboratory models to simulate the evolutionary process (Taylor et al. 1983; Lopes et al. 2008; Busi and Powles 2009). Previous experimental evolution studies have reported rapid evolution of drug resistance in a variety of organisms; including insects, nematodes, and other invertebrates (Barros et al. 2001; Lopes et al. 2008; Jansen et al. 2011). These studies often employ one of two strategies in generating resistance: (i) impose a continuous drug or pesticide dose on a population and monitor adaptation over a number of generations; or (ii) increase drug dose at regular intervals, often every generation, to track the dose of drug required to cause a target mortality level (e.g. 50% mortality; LD50) in the population under selection. Few studies have specifically looked at the effect of dosage on the rate of resistance evolution, although Busi and Powles (2009) found that selection under exposure to both low and high doses of glyphosate caused a rapid increase in survival of rye grass over three generations and that higher doses promoted a greater magnitude of resistance. However, resistance screens were performed on the first generation offspring of selected plants, therefore any response could have been due to maternal effects. Experimental selection over multiple generations at different sublethal doses would help to further elucidate the relationship between dose and the rate of resistance evolution.

In addition to dosage, differences in population density between treated lines of parasites and pests could result in differential selection due to density‐dependent processes such as competition (Gilleard and Beech 2007). Laboratory‐based selection experiments often impose strong selection on generation time or timing of reproduction when reproductive strategies are influenced by density‐dependent effects (Chehresa et al. 1997). Since the application of a drug or pesticide treatment reduces population size, this will create differences in population density between treatments, which could alter the apparent evolution of resistance due to changes in traits that are not directly associated with responding to chemical exposure (Gilleard and Beech 2007). Selection experiments investigating the rate of resistance evolution typically involve comparisons of survival and/or life history in a drug treatment compared to a control treatment with no drug applied (Ranjan et al. 2002; Coles et al. 2005; Lopes et al. 2008). However, this methodology does not account for differences in population density resulting from differences in mortality between the treatments. If studies are to be biologically realistic and drug treatments involve the bottlenecking of populations, then the experimental design must separate the indirect effects of reduced density from the direct effects of the drug (Fuller et al. 2005).

The treatment of helminth diseases provides a well‐documented field of research in which to explore problems related to resistance evolution using an experimental approach (Driscoll 1989; Sangster and Gill 1999; Kaplan and Vidyashankar 2011). Ivermectin is a broad‐spectrum antiparasitic drug and has been used commercially since 1981 (James et al. 2009), with the first reports of resistance in 1988 (Kaplan 2004). Ivermectin causes paralysis in larvae and adult nematodes and inhibits feeding (Sangster and Gill 1999), but also has a repellent effect at sublethal doses (Ardelli et al. 2009). Because parasitic helminths are difficult to culture, research into anthelmintic resistance has a long history of using the model organism *Caenorhabditis elegans* in both drug screening and identifying candidate resistance loci (Simpkin and Coles 1981; James et al. 2009; Ghosh et al. 2012). However, *C. elegans* is an androdioecious nematode species that reproduces mainly by self‐fertilization, although low levels of outcrossing do occur as a result of the small proportion of males present in a population (Brenner 1974; Barrière and Félix 2007). Since most parasitic nematodes are dioecious and obligately outcrossing, other free‐living dioecious nematodes such as *Caenorhabditis remanei* may provide a more realistic model system to explore resistance evolution. *Caenorhabditis remanei* populations have abundant standing genetic variation and high levels of recombination due to their reliance on sexual reproduction (Cutter et al. 2006). Both of these attributes should facilitate a rapid response to selection. Additionally, *Caenorhabditis* species provide an effective microcosm system, which has been used to answer a broad range of evolutionary questions related to rapid evolutionary change (Lopes et al. 2008; Morran et al. 2011; Gray and Cutter 2014). Manipulating drug dosage, as well as controlling for differences in population density between treated lines in simple microcosm systems, may provide us with a better understanding of how natural populations of parasites and pests adapt to control measures.

The terms resistance and tolerance are often used interchangeably when defining reduced susceptibility to xenobiotics and has led to much confusion on their relative importance in the evolution of reduced susceptibility. Tabashnik et al. (2014) define resistance as a genetically based decrease in susceptibility as a result of exposure to a control agent; this definition emphasizes a heritable change in susceptibility of a target population due to previous exposure to a control measure. In other words, the spread of resistance through a population is the result of an increase in frequency of pre‐existing alleles conferring reduced susceptibility, novel or spontaneous mutations or migration of resistance alleles between populations during a period of time where the population is exposed to a drug (Gilleard and Beech 2007). By this definition, a population cannot be resistant prior to exposure to a control agent and resistance results as an evolved response, specifically due to drug application. Tolerance, on the other hand, is due to natural variation in susceptibility already pre‐existing within or between populations rather than a result of selection pressure imposed by control measures (Scott 1995). Tolerance may also be used to describe pre‐existing differences in susceptibility between different species or between life‐history stages of organisms (Coles and Dryden 2014). For example, sensitivity to Ivermectin has been shown to vary substantially among species of sepsid dung flies (Puniamoorthy et al. 2014). Puniamoorthy et al. (2014) found that tolerance was explained by phylogenetic relationships; more closely related species had similar levels of susceptibility to Ivermectin on naive exposure. However, they could not rule out the possibility of rapid adaptation of species to Ivermectin but suggested that this was unlikely as they found more variation in Ivermectin sensitivity between species within sample sites than variation within species between sample sites. Additionally, some of the least susceptible species were known to be drug naive as they were sampled from locations where anthelmintics have not been used. This suggests that tolerance may occur due to pleiotropic effects and selection on some other unknown trait may result in pre‐adaptation in the form of reduced susceptibility. If the frequency and magnitude of tolerance within a population is affected by selection on unknown traits, the factors which effect selection on those traits will play an important role in governing susceptibility to control agents prior to exposure. In addition, drug‐treated populations could evolve tolerance in parallel to resistance if evolved decreases in susceptibility are associated with density‐dependent selection, and affect the apparent rate of resistance evolution (Gilleard and Beech 2007). It is difficult to separate tolerance from resistance unless this is explicitly incorporated into the experimental design but this also requires knowledge about which traits confer differences in tolerance to a particular xenobiotic.

The overall aim of this study was to assess how Ivermectin dosage, and changes in population density affect the rate of resistance evolution in replicate lines of *C. remanei*. Specifically, we asked: (i) What is the relationship between *C. remanei* survival and Ivermectin dose over a range of concentrations within a single generation? (ii) Is there an increase in survivorship across generations of populations selected in drug‐treated environments, and does this vary with dosage? (iii) Does density‐dependent selection affect the apparent evolution of resistance in selected lines? (iv) Is there a cost of adaptation to drug‐treated environments in terms of survival in drug‐free environments? We also explored the relationship between life‐history and drug selection, asking: (v) Does survival of different life‐history stages (juvenile and adult) respond to drug‐selection in the same way?

## Methods

### Origin and maintenance of experimental lines

In order to maximize the degree of standing genetic variation available to select for resistance, we obtained a genetically diverse strain of *C. remanei* (SP8) from N. Timmermeyer in the Department of Biology, University of Tübingen, Germany. This strain was originally created by a fully factorial crossing of three wild‐type strains isolated from geographically distant locations (SP146 from Freiburg, Germany; MY31 from Tübingen, Germany; PB206 Ohio, USA). Crosses had been tested for fertility, offspring pooled, and maintained for eight generations to create recombinant genotypes and allow adaptation to standard laboratory conditions (Fritzsche et al. 2014). Upon arrival in Glasgow, strain SP8 spent a further four generations adapting to any differences in conditions between laboratories and was maintained under standard laboratory conditions for *Caenorhabditis* species: 20°C and 60% humidity on NGM (Nematode growth medium) petri dishes and fed on a lawn of *Escherichia coli* (OP50) (Hope 2001).

### Dose–response assay

In order to choose two distinct doses that differ in the intensity of selection imposed during the selection experiment, it was first necessary to quantify the relationship between drug dosage and survivorship for strain SP8. A stock solution of 2 mg/mL Ivermectin (22,23‐Dihydroavermectin B1; Sigma‐Aldrich) dissolved in DMSO was decanted into 1 mL aliquots and frozen to provide a standardized drug dose. We used a modified version of the dose–response approach taken by Rufener et al. (2010) to quantify survivorship of *C. remanei* over a range of doses (0, 0.1, 0.5, 1, 1.5, 2, 2.5, 3, 4, 5, 6, 7, 8, 9, and 10 ng/mL). Appropriate dilutions of Ivermectin were administered to 100 mL liquid NGM (50°C) and mixed with a magnetic stirrer before pouring 7 mL aliquots into 5.5 cm plastic petri dishes. These were left to dry, seeded with *E. coli* (OP50) *ad libitum* to minimize indirect mortality resulting from repellence at low doses and incubated at 20°C overnight. Concurrently to preparing dosed plates, age‐synchronized eggs were harvested from stock populations of *C. remanei* by bleaching using standard protocols. This process kills adults and juveniles but leaves developing embryos unharmed (Hope 2001). Eggs were moved to fresh 9 cm drug and food‐free petri dishes and incubated overnight to provide a source of L1‐arrested larvae for drug screening. After 12 h incubation, larvae were suspended in M9 buffer solution (3 g KH_2_PO_4_, 6 g Na_2_HPO_4_, 5 g NaCl, 1 mL 1 m MgSO_4_, H_2_O to 1 L and sterilized by autoclaving) and 5 μL aliquots of this suspension were added to Ivermectin‐dosed plates with the aim of applying approximately 60 larvae per plate. Larvae added to petri dishes were counted as they were set‐up; survival data were obtained by counting the number of adults present per plate at 75 h. *Caenorhabditis remanei* become reproductively active 2 days after hatching (Diaz et al. 2008) so survivorship was measured at 75 h after L1 larvae were exposed to the relevant dose of Ivermectin. Twenty replicate plates were established for each Ivermectin dose (ten replicates in each of two different batches conducted at different times).

### Selection experiment

**Figure 1:**
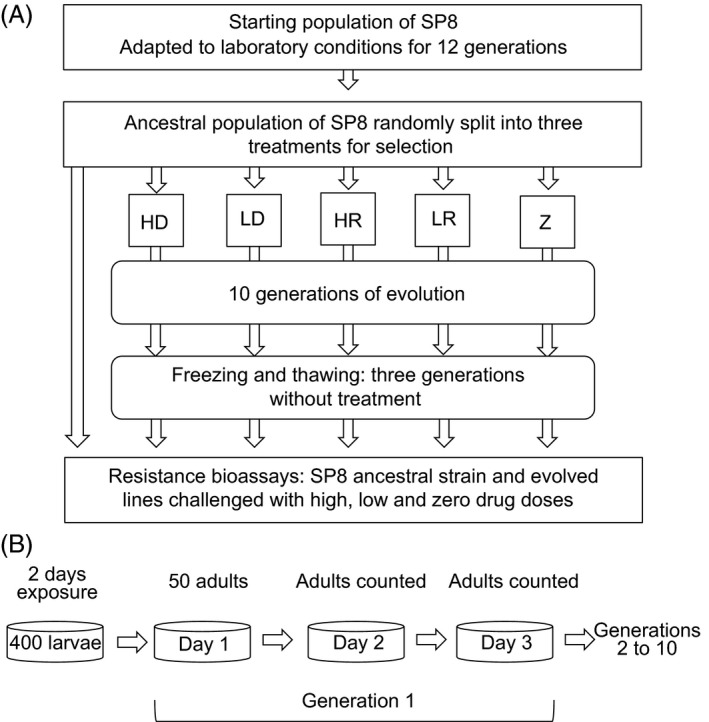
(A) Schematic representation of dose–response assay, selection experiment, and resistance bioassay. The starting population of SP8 was adapted to laboratory conditions. The laboratory‐adapted strain was then assayed for variation in susceptibility to Ivermectin over a range of 15 doses, to select an appropriate high and low dose for the selection experiment. The laboratory‐adapted strain was then randomly divided into five treatments with three replicates each for HD, LD, HR, and LR lines, and six replicates for Z lines. After 10 generations of selection, lines were frozen and later thawed, before being challenged with the three doses of Ivermectin used during the original selection experiments. (B) Schematic representation of selection experiment showing initial population set‐up and one generation. Initially, lines were established with 400 larvae exposed to the relevant dose of Ivermectin; 50 adults were then selected to begin generation 1 on day 1. After 24 h lines were counted and compensatory mortality imposed on random lines; this was at 48 h. After 72 h, subadults from the next generation were transferred to new plates. Generations 2–10 proceeded as for generation 1. HD, high‐dose treatment; HR, high‐random treatment; LD, low‐dose treatment; LR, low‐random treatment; Z, zero dose treatment.

Two Ivermectin doses were chosen as drug treatments for experimental evolution (Figure S1B): (i) a high dose that corresponded to 80% mortality at 75 h in naive populations; and (ii) a low dose that corresponded to 40% mortality. These two doses were combined with a control of no drug application (zero = Z, low drug = LD, and high drug = HD, Fig. 1A). In addition, a random‐mortality treatment was included for the low and high dosages to account for differences in density between drug treatments (low random = LR, and high random = HR) by randomly removing the same number of individuals from these plates as had died in response to the corresponding drug treatment. For instance, if two females and six males had died in a drug‐treated line, a sister random‐mortality line had the same number of each sex removed. All lines were exposed to high (HD and HR) and low mortality environments (LD and LR), with three replicates per experimental line per treatment, with the exception of the controls, which were replicated six times.

Experimental lines were cultured for 10 generations. The ancestral stock strain (generation 0) as well as samples of larval worms from each line at generations 5 and 10 were cryogenically frozen at −80°C Fig. 1A), at a density of approximately 2000 L1 larvae in liquid freezing solution as described in Hope (2001). Generation 1 (18 lines overall) was initiated using standard bleaching methods from the ancestral stock strain of SP8 cultured in the lab for four generations after thawing and represents the ancestral condition (generation 0; Fig. 1B). L1‐arrested larvae were suspended in M9 buffer and worm density of the suspension obtained by counting worms from five replicates of 5‐μL aliquots. A volume of the suspension corresponding to 400 L1‐larvae was then added to *E. coli* seeded NGM plates (9 cm) with the appropriate dose of Ivermectin. Establishing populations with 400 larvae prevented density‐dependent competition but still contains sufficient numbers of individuals to ensue a substantial proportion of standing genetic variation (Allendorf 1986). After 48 h of development worms reach the 4th larval stage (L4) at which point the sex can be determined. At this time, 25 pairs of male and female L4 larvae were transferred to fresh agar plates of the appropriate dose for each replicate. These 50 adults constituted generation 1, day 1. After 24 h, adults were counted and census data were used to impose an equivalent mortality on the random‐mortality lines for the respective treatments. After 48 h of drug exposure, the same process of adult census and compensatory‐induced mortality was repeated. By 72 h, larvae from the next generation had developed to L4 larvae: 25 pairs were selected to continue the next generation and transferred to fresh petri dishes. This was continued for 10 generations. Census data were gathered each generation to assess whether there was an increase in survivorship of lines selected in drug‐treated environments and whether this increase varied with dosage. In addition to adult census, a juvenile census was performed after 48 h to provide an estimate of juvenile population densities. L2 and L3 larval stages were counted along a 1 cm transect covering the center of the petri dish; L1 juveniles were too small and numerous to gather reliable counts.

### Drug‐resistance bioassays

In order to formally assess whether heritable increases in survivorship were imposed by selection with Ivermectin, ancestral stocks (generation 0) as well as each of the selected lines from generation 5 and 10 were exposed to the same high and low doses of Ivermectin used during selection and raised in a drug‐free environment. Firstly, to test the effects of drug dosage on survival, revived samples of HD, and LD lines were exposed to a dose of Ivermectin corresponding to that used during selection. Survival of these lines was then contrasted with survival of Z lines to assess whether there was a change in evolved lines. Secondly, to test for effects of differences in population density on survival of selected lines, we exposed HR and LR lines to a high and low dose of Ivermectin, respectively. Survival of HR and LR lines were contrasted with Z lines, with any significant differences in survival between random mortality and Z lines indicating an effect of population density on relative survival. Thirdly, we tested for any cost to adaptation to selection regime in terms of survival by raising evolved lines in a drug‐free environment, with the hypothesis that if there is a cost to adaptation, then experimentally treated lines should show significantly lower survival than control (Z) lines.

Preserved samples of lines from the selection experiment at generations 0, 5, and 10 were thawed and raised for three generations in a drug‐free environment to ensure that any observed responses in survival were due to genetic differences among populations and not maternal or environmental effects due to freezing. Larvae were thawed at room temperature and maintained at a density of approximately 1000 individuals per 9 cm agar plate over the three generations from thawing to age synchronization with *ad libitum* lawns of *E. coli* OP50. Transfers between generations were achieved by cutting out sufficient agar from plates already containing samples and transferring these to fresh *E. coli* seeded plates ensuring the density remained as constant as possible. Agar plates, synchronization of experimental lines and set‐up of larvae were conducted with the same protocol used in the dose–response assay. Mortality due to drug application may differ between life‐history stages; in order to gain some measure of this difference, we measured survival both at 52 h, encompassing juvenile development and 75 h, during the first day of reproduction. Generations 5 and 10 of each experimental line were replicated four times, as was the ancestral line (generation 0).

### Statistical analyses

All statistical analyses were performed using r v 3.1.2 (R Core Team 2014) and we defined a significance threshold of *P* = 0.05 for all tests. A more detailed description of the rationale for the statistical approaches used is provided in the Supporting information. The doses required to cause 40% and 80% mortality of the ancestral SP8 strain were estimated, with 95% CI's, using the drc package (Ritz and Streibig 2007). In order to calculate estimates of these two doses, we constructed a dose–response curve of the relationship between worm survival and concentration of Ivermectin. We fitted a range of dose–response models (log‐logistic, Weibull‐1, and Weibull‐2) with the lower asymptote of the curve fixed at 0% survival and used maximum likelihood to select the most appropriate model of survival data. Ivermectin concentration and batch were fitted as fixed effects in our full model. To assess whether the relationship between survivorship and Ivermectin concentration remained the same between batches performed at different times (i.e. repeatability), batch was removed from the model and compared against the full model using a likelihood ratio test. Estimates of the required doses, with 95% CIs, were then derived from model predictions.

Our experimental design incorporated a power analysis, which specifically adjusted for the effects of the number of lines, interline variation, the potential observable difference in survival between treatments (effect size), and bioassay replicate (Johnson et al. 2015). We estimated that our experimental design gave 93% power to detect an absolute difference in survivorship of 10% in the high‐dose environment between the control Z lines and both HD and HR lines. To assess whether survivorship changed over the course of the selection experiment, data from the resistance bioassay were analyzed using generalized linear mixed models using the glmer function in the lme4 package assuming a binomial error distribution with a logit link function (Bates et al. 2014; see Supporting information). Treatment and generation and the interaction between them were fitted as fixed effects. The evolutionary replicate (line) was fitted as a random effect. An observation‐level random effect was fitted to account for any overdispersion between replicate lines in the selection experiment and repeated sampling of populations in the drug resistance bioassay (Browne et al. 2005). Treatment effects in the selection experiment were tested using likelihood ratio tests. The null hypothesis of no difference in survival between the three treatments (*H*_0_: Drug = Random = Zero) was tested independently for high‐ and low‐mortality selection regimes by comparing the full model with a null model with no fixed effect of treatment or interaction terms. Generation was kept in the null model to account for any drift in survivorship. Three posthoc tests comparing treatment pairs were then conducted to assess the effects of individual treatments. This general approach was used to answer each of our research questions.

## Results

### What is the relationship between *Caenorhabditis remanei* survival and Ivermectin dose over a range of concentrations within a single generation?

Two Ivermectin doses were chosen as drug treatments for experimental evolution (Figure S1B): (i) a high dose that corresponded to 80% mortality in the stock strain at a concentration of 2.46 ng/mL Ivermectin (95% CI: 2.41, 2.50); and (ii) a low dose that corresponded to 40% mortality at 75 h at a concentration of 1.61 ng/mL Ivermectin (95% CI: 1.55, 1.68). Analysis using comparisons of log likelihood found that a three‐parameter Weibull‐1 model with the lower asymptote fixed at zero gave the best fitting model of survival as a function of the concentration of Ivermectin (Figure S1A) and there was no difference between the two survival curves for data collected in the two batches (*χ* ^2^ = 6.821, df = 3, *P* = 0.0778; Figure S1A).

### Is there an increase in survivorship of populations across generations selected in a drug‐treated environment, and does this vary with dosage?

**Figure 2:**
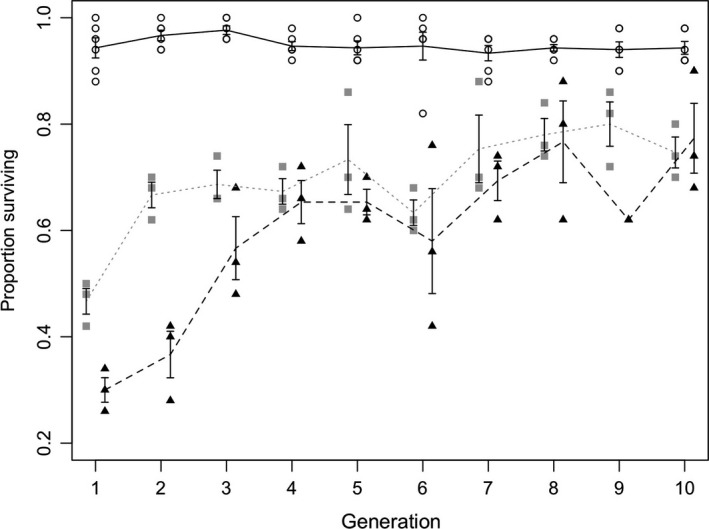
Survivorship during original selection experiments. Lines represent mean survival for each treatment; points are the proportion of adults surviving on day 2 of each generation for each replicate line within a treatment. Circles, solid line = zero dose; squares, dotted line = low dose; triangles, dashed line = high dose. Error bars; standard error for mean survival.

In the selection experiments (Fig. 2), survival in zero‐dose populations remained constant over generations; the mean adult survival in generation 1 was 94% (CI: 90%, 99%), at generation 5 survival was 94% (CI: 90%, 98%) and at generation 10 survival was 94% (CI: 91%, 97%). Larval offspring densities of zero‐dose lines also remained relatively constant over the course of 10 generations; mean larval density at generations 0, 5, and 10 was 2079, 2051, and 1878 respectively (Figure S2). In lines treated with the lower dose of Ivermectin, survival increased gradually over 10 generations, from 47% in generation 1 (CI: 36%, 57%) to 73% (CI: 45%, 100%) at generation 5 and 75% (CI: 62%, 87%) in generation 10. Larval offspring numbers remained low in LD lines throughout the course of the selection experiment; the mean number of offspring at generation 1 was 1088 at generations 5 and 10 it was 1132 and 1248 respectively (Figure S2). Survival in high‐dose treated populations increased more dramatically, from 30% (CI: 20%, 39%) at generation 1, to 65% (CI: 54%, 76%) at generation 5 and 77% (CI: 49%, 100%) at generation 10. Offspring numbers of HD lines increased during the selection experiment; the mean number of offspring was 394, 1166 and 1435 at generations 0, 5 and 10 respectively (Figure S2).

**Figure 3:**
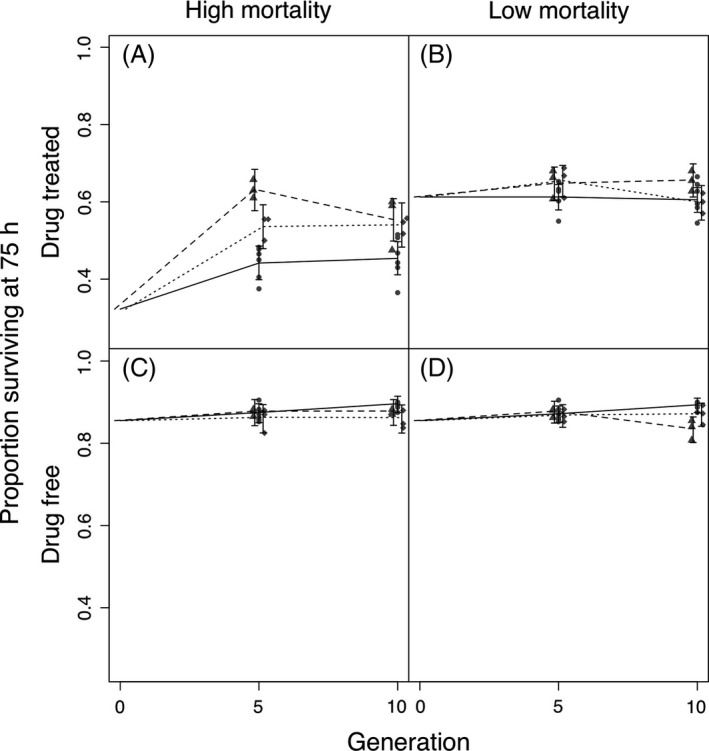
Seventy‐five hour survival when exposed to the three drug doses used during selection (A = high; B = low: C and D = zero) of samples taken from generations 0, 5, and 10 during selection. (A, C) Survivorship of high mortality lines: HD and HR. (B, D) Survivorship of low‐mortality lines: LD and LR. Points are mean survival data for each replicate population, lines represent predictions of maximal models (generation + treatment + generation*treatment) for each treatment: circles, solid line = zero dose; triangles, dashed line = drug treatment; diamonds, dotted line = random mortality. Error bars; 95% confidence intervals for mean survival. HR, high random; LR, low random.

In our formal test of changes in susceptibility of evolved lines, challenge with the dose used during selection, HD lines exposed to a high dose of Ivermectin for 75 h exhibited an increase in mean survival of 19% and 10%, at generations 5 and 10 respectively, relative to Z lines (*H*_0_: HD = Z: *P* < 0.0001; Fig. 3A, Table 1). Survival was relatively consistent between lines within a treatment (Figure S3). Mean survivorship of the three HD lines remained between 59% and 66% at both generations 5 and 10, except in the case of one line in generation 10 where survivorship dropped to 48%. Variation in the mean survivorship of the six Z lines ranged between 37% and 51% at both generations 5 and 10. At 52 h of exposure to Ivermectin, the HD lines showed a similar increase in mean survival to data collected at 75 h (Table S1, Figure S4A). Thus, both juveniles and adults exhibited a comparable response to selection in terms of increased survival in the high‐dose environment. LD lines exposed to a low dose of Ivermectin for 75 h showed no increase in survival relative to control lines (*H*_0_: LD = Z: *P* = 0.11; Fig. 3B, Table 1), but at the earlier observation time of 52 h LD lines exhibited increased survival relative to Z lines at both generations 5 and 10 (*H*_0_: LD = Z: *P* = 0.022; Table S1, Figure S4B). Therefore, selection at the low dose of Ivermectin resulted in higher survivorship of juveniles but not adults when re‐exposed to a low‐drug dose.

**Table 1:** Effect of treatment during selection (mortality treatment) on survivorship (Surv.diff) after 75 h, in drug‐treated environments (dose); assessed by null models (see Data S1), using likelihood ratio tests, where survival is constrained to be equal across treatments, and dependent upon the best fitting model

Mortality treatment	Dose	Best fitting model	Null models	*χ* ^2^ (df)	*P*‐value	Surv.diff	
						Gen 5	Gen 10
High	High	G + T + G × T	1. HD = HR = Z	22.26 (4)	0.00018		
			2. HD = Z	21.11 (2)	<0.0001	0.19	0.10
			3. HR = Z	8.56 (2)	0.014	0.09	0.09
			4. HD = HR	6.56 (2)	0.038	0.10	0.01
	Zero	G	1. HD = HR = Z	3.59 (2)	0.47		
Low	Low	G	1. LD = LR = Z	7.67 (2)	0.11		
	Zero	G + T + G × T	1. LD = LR = Z	11.47 (4)	0.022		
			2. LD = Z	11.33 (2)	0.0035	−0.01	−0.06
			3. LR = Z	1.84 (2)	0.40	0.00	−0.02
			4. LD = LR	3.25 (2)	0.20	−0.01	−0.04

G, generation; T, treatment; G × T, generation × treatment interaction; HD, high‐dose treatment; HR, high random treatment; LD, low‐dose treatment; LR, low random treatment; Z, zero‐dose treatment; df, degrees of freedom; Surv.diff: absolute difference in mean survival between the highlighted treatments (first minus second).

### Does density‐dependent selection affect the apparent evolution of resistance in selected lines?

In the selection experiment, survival in both random mortality treated lines remained of a similar magnitude to zero‐dose lines prior to random removal of worms; the mean adult survival of LR lines at generation 1 was 95% (CI: 92%, 98%), at generation 5 survival was 97% (CI: 86%, 100%) and at generation 10 survival was 93% (CI: 90%, 96%). Larval densities of LR lines remained similar to those of LD lines during the selection experiment; the mean number of larvae was 1088, 1132, and 1248 at generations 0, 5, and 10 respectively (Figure S2). Mean adult survival of HR lines at generation 1 was 96% (CI: 91%, 100%), at generation 5 survival was 96% (CI: 87%, 100%) and at generation 10 survival was 95% (CI: 87%, 100%). Offspring numbers of HR lines during the selection experiment remained lower than zero‐dose controls; the mean number of offspring was 1083, 1203, and 1172 at generations 0, 5, and 10, respectively (Figure S2).

Surprisingly, in the resistance bioassays, high random mortality (HR lines) showed an increase in mean survival when populations were challenged with a high dose of Ivermectin. Mean survival of HR lines was 9% higher than Z lines for both generations 5 and 10 after 75 h (*H*_0_: HR = Z: *P* = 0.014; Fig. 3A, Table 1). Therefore, reducing density by removing individuals randomly had a similar effect to drug treatment in HD lines. However, there was a difference between HD and HR treatments; HD lines showed higher survival at generation 5 but not 10 (*H*_0_: HD = HR: *P* = 0.038; Fig. 3A, Table 1). Variation in mean survivorship of the three HR lines remained consistently between 50% and 56% at both generations 5 and 10; smaller than the between‐line variation observed in both HD and Z lines (Figure S3). At 52 h of drug exposure, the increase in survival of HR lines relative to Z lines was comparable to that of data collected at 75 h (Table S1, Figure S4A). Thus, when exposed to the high dose of Ivermectin, survival of both juveniles and adults from HR lines responded to selection in a similar manner. Survivorship of lines selected in the LR environment showed no response to selection when exposed to a low dose of Ivermectin for 75 h; survivorship remained comparable to that of Z lines at both generations 5 and 10 (*H*_0_: LD = LR = Z: *P* = 0.11; Fig. 3D, Table 1). However, when survival of LD lines was observed at 52 h of exposure to a low‐drug dose, survival was similar to LD lines, relative to Z lines (*H*_0_: LR = Z: *P* = 0.035; Figure S4A, Table S1). As was the case with LD lines, increased survivorship of LR lines in the low‐dose environment was only observed for juveniles at 52 h, and not adults at 75 h.

### Is there a cost of adaptation to drug‐treated environments in terms of survival in drug‐free environments?

In an environment where no drug was administered, HD and HR lines performed equally as well as Z lines in terms of survival over 75 h (*H*_0_: *χ* ^2^ = 3.95, df = 2, *P* = 0.47; Fig. 3C, Table 1). In contrast, LD lines had significantly lower survivorship than Z lines in the drug‐free environment. However, this was only apparent at generation 10 and the magnitude of the effect was relatively small (*H*_0_: LD = Z: *P* = 0.0035; Fig. 3D, Table 1). LR lines also maintained a similar response in survivorship as Z lines at both generation 5 and 10 (*H*_0_: LR = Z: *P* = 0.40; Fig. 3D, Table 1), and there was no significant difference between LR and LD lines with respect to survival (*H*_0_: LD = LR: *P* = 0.20; Fig. 3D, Table 1). The relationship in survival measurements taken at 52 h for the evolved lines remained similar to survival measured at 75 h for all treatments (Table S1, Figure S4C,D).

### Does survival of different life‐history stages (juvenile and adult) respond to drug‐selection in the same way?

Mortality due to drug challenge continued between 52 and 75 h in HD and LD selected lines when challenged with Ivermectin and was of a greater magnitude than observed in a drug‐free environment (Fig. 3 and Figure S4). When exposed to the dose used during selection, HD lines showed no interaction between generation and selection regime at 52 h (*χ* ^2^ = 1.33, df = 2, *P* = 0.51) but at 75 h an interaction was apparent (*χ* ^2^ = 5.96, df = 2; *P* = 0.05). The change in significance of treatment and generation interactions indicates a change in the way juvenile and adult survival responded to drug selection in HD lines; juvenile survival remained similar between generations 5 and 10, whilst adult survival declined (Fig. 3A and Figure S4A). When worms were exposed to a low dose of Ivermectin, we observed differential survival between LD and control (Z) lines at 52 h but not at 75 h (Fig. 3B and Figure S4B, Table 1); suggesting that juvenile survival responded to drug selection but adult survival remained unaffected by drug treatment.

In our pooled data sets, we found no evidence of a three‐way interaction between selection experiment treatment, bioassay dose and life‐history stage at generations 5 or 10 (*χ* ^2^ = 2.77, df = 4, *P* = 0.60, *χ* ^2^ = 0.47, df = 4, *P* = 0.98, respectively). However, there was a significant two‐way interaction between selection experiment treatment and bioassay dose at both generations 5 and 10 (*χ* ^2^ = 28.98, df = 4, *P* < 0.0001, *χ* ^2^ = 38.96, df = 4, *P* < 0.0001, respectively); suggesting that survival in drug‐treated environments was dependent on selection regime. In addition, there was an interaction between bioassay dose and life‐history stage at generation 5 but not generation 10 (*χ* ^2^ = 6.07, df = 2, *P* = 0.048, *χ* ^2^ = 3.82, df = 2, *P* = 0.15, respectively). There was no evidence of an interaction between selection experiment treatment and life‐history stage for generations 5 or 10 (*χ* ^2^ = 0.77, df = 2, *P* = 0.68, *χ* ^2^ = 4.40, df = 2, *P* = 0.11, respectively).

## Discussion

### What is the relationship between *Caenorhabditis remanei* survival and Ivermectin dose over a range of concentrations within a single generation?

The dose–response curve of the survival of the drug‐naive ancestral strain of *C. remanei* (SP8) was similar to those previously reported for drug‐naive *C. elegans* when challenged with a range of Ivermectin concentrations (James and Davey 2009). The confidence intervals of the two Ivermectin doses used in the selection experiment differed; the high dose had narrower intervals than the low dose. This suggests that the intensity of selection applied to the first generation of the selection experiment was more variable in lines exposed to low doses of Ivermectin, though even at low doses this would translate into no more than ±3.25% variation in survival.

### Do drug‐treated lines show an increase in survivorship across generations in drug‐treated environments, and does this vary with dose?

Census data from the selection experiment indicated that populations of *C. remanei* exposed to low and high doses of Ivermectin showed a response to selection in terms of increased survival over 10 generations (Fig. 2, Table 1). Furthermore, the increase in survivorship in HD lines was of a greater magnitude than LD lines, suggesting that evolution was more rapid in populations exposed to a higher drug dose. The data from resistance bioassays support the responses observed in the selection experiment in terms of the greater magnitude of response in survivorship of HD lines relative to LD lines. In both dosage regimes, the increase in survivorship during the selection experiment slowed over the course of the experiment, suggesting a rapid response of populations to drug treatment that reached a peak for a given drug dose. Rapid responses to drug selection and peaking of the response have been previously observed in Levamisole‐selected strains of *C. elegans* (Lopes et al. 2008).

Previous research focused on under‐dosing has suggested that lower doses (doses below recommended use) may promote the evolution of resistance, especially where the basis of resistance is polygenic (Manalil et al. 2011; Shi et al. 2013), and that varying the level of under‐dosing may affect the rate at which resistance evolves (Busi and Powles 2009). Our data suggest that selection at a low dose of Ivermectin conferred no advantage on LD lines when re‐exposed to the low‐dose environment for 75 h. However, HD‐selected lines showed higher survivorship relative to Z lines on exposure to the high‐drug dose. Thus, the intensity of selection played a role in how selected populations responded to Ivermectin treatment. The lack of a response in survival of LD lines exposed to the low dose for 75 h conforms to models of resistance evolution in nematodes where under‐dosing retards the development of resistance (Barnes et al. 1995). Under such models under‐dosing may reduce the evolution of resistance by allowing more susceptible worms to survive.

### Does density‐dependent selection affect the apparent evolution of resistance in selected lines?

Intriguingly, survival of lines selected in random‐mortality environments showed a similar trend, but of a lower magnitude, to drug‐selected lines, and in contrast to zero‐dose lines, suggesting that density‐dependent effects on life‐history traits might be affecting the apparent rate of resistance evolution. Random culling of adults reduced larval densities in random mortality treated lines; meaning that larval densities remained comparable to drug‐treated lines and lower than control (Z) lines. Density‐dependent natural selection has been shown to affect the competitive abilities of selected lines; Mueller (Mueller 1988) showed that the feeding efficiency of *K*‐selected (high density) lines was 58% greater than *r*‐selected (low density) lines of *Drosophila melanogaster* after 128 generations of density‐dependent selection. Though our selection experiment design aimed to provide an abundant bacterial food source, at the time lines were transferred to new plates, bacterial lawns were patchy and no doubt some competition for resources is likely to have occurred. Life‐history traits such as development time, size at maturity and reproduction may all be influenced by density‐dependent selection (Joshi et al. 2001; Prasad and Joshi 2003; Dey et al. 2012). If traits associated with selection in a low‐density environment confer an advantage in a novel drug‐treated environment, then this may explain the observed increase in survivorship of random‐mortality lines relative to control (Z) lines. Thus, much of the observed response in survivorship in drug‐treated and random‐mortality lines when challenged with Ivermectin could be due to increased tolerance as a result of density‐dependent processes, rather than resistance evolution *per se*. Put another way, if the response in survival of HR lines is attributable to the evolution of tolerance then perhaps a large part of the response in survival of HD lines, which would have faced similar density‐dependent processes to HR lines, is also due to selection for tolerance rather than resistance.

Alternatively, the increase in survivorship of drug‐treated and random‐mortality lines when exposed to drug treatment could be a result of loss of genetic variation due to drift. This hypothesis would require all lines to drift in the same direction, which could have occurred during bottlenecking of drug‐treated and random‐mortality lines, particularly in the early generations of selection. However, the loss of diversity may not have been severe relative to the control zero‐dose lines (see Data S1: drift and loss of diversity). Our theoretical predictions of the loss of genetic diversity in HR and Z lines suggest that both treatments went through similar losses of genetic diversity. Predicted heterozygosity and the total number of alleles decreased more rapidly in HR lines relative to Z lines but the difference between the two treatments was small. In the case of rare alleles, it is likely that any rare allele would have been lost from populations in both HR and Z lines. Thus, it seems likely that any evolved increase in survivorship of HR and potentially drug‐treated lines, was due to ecological processes occurring as a consequence of density‐dependent selection and not loss of genetic variation due to drift.

Differentiating between the effects of drug selection and traits not directly associated with resistance has been a long‐standing problem in studies of resistance evolution (Chehresa et al. 1997; Gilleard and Beech 2007). The increase in survival of HR lines over generations when challenged with both low and high drug doses was of a lower magnitude than HD lines; this difference in absolute survival could represent the effects of selection solely due to drug treatment. If this is the case, then our experimental design provides a means of partitioning the evolved response in survival due to drug application and responses due to the effects of population size, density, and the risk of mortality. Increased parasite densities generally have a negative effect on traits such as survival and fecundity (Churcher et al. 2006); however, how density‐dependence interacts with drug treatment remains unclear and may depend upon which life‐history stage is most severely affected by the drug (Churcher and Basáñez 2008). It is also possible that the difference in survivorship between HR and HD lines was due to the experimental protocol during selection. Random‐mortality populations were culled once every 24 h to simulate the same level of mortality as ‘sister’ drug‐treated populations, but drug‐treated populations are likely to have suffered additional mortality over the course of this 24‐h period. This would have resulted in a lag between drug‐induced mortality and culling between ‘sister’ populations. If HR lines had tracked the rate of mortality in HD lines more closely, maintaining similar densities between HD and HR treatments, potentially the same magnitude of response could have been observed in both high mortality treatments, regardless of mortality source. A more synchronized method of tracking drug‐dependent mortality and imposing compensatory mortality on random‐mortality lines would reveal whether the lag in random culling is responsible for the difference in survivorship between HR and HD lines.

### Is there a cost of adaptation in drug‐treated environments in terms of survival in drug‐free environments?

When random‐mortality and drug‐treated lines were exposed to a drug‐free environment, no differences were observed in survivorship relative to Z lines. Therefore, bottlenecking and small population size of random‐mortality lines resulted in no beneficial or detrimental effects on survival in an environment where no extrinsic mortality was imposed. It has been suggested that the evolution of reduced susceptibility may lead to fitness costs in life‐history traits if resistance is costly (Roush and McKenzie 1987). In order to assess the fitness costs that might result from reduced susceptibility one could either measure gene frequencies of susceptible alleles over a number of generations in the absence of the drug (Roush and McKenzie 1987) or estimate fitness based on measures of life‐history traits such as fecundity, development time, fertility and mating competitiveness (Carriere et al. 1994; Gassmann et al. 2009) in the presence and absence of the drug. In this study, we looked solely at differences in survival in drug‐free and drug‐treated environments; it would be interesting to assess a suite of traits associated with fitness and explore their relationship with apparent susceptibility to Ivermectin.

### Do different life‐history stages respond to drug selection in the same way?

Mathematical models have suggested that the life history of parasites may evolve in response to drug‐treatment as a result of altering parasite survival and reproduction (Lynch et al. 2008). The differing responses of life‐history stage (juveniles and adults) in HD and LD lines at low and high dosages suggest that age‐related effects and interactions with selection intensity may be important to consider in predicting resistance or tolerance evolution. We observed a significant interaction between resistance bioassay dose and life history. In addition, resistance bioassay data from 75 h showed a response in survivorship of HD lines but not LD lines; i.e. adults of HD lines were less susceptible than Z lines whereas LD lines remained of a similar susceptibility to Z lines, across selected generations. However, 52‐h bioassay data showed that both HD and LD lines responded to drug selection in terms of increased survivorship. Therefore, at the high dose of Ivermectin, both juveniles and adults responded to drug selection, whereas at low doses only juveniles responded to selection.

Body size is often used as a predictor of fecundity across a range of nematode species (Morand 1996). Under standard life‐history theory, interventions that reduce adult life expectancy should select for parasites that mature earlier at a reduced size and produce fewer offspring (Roff 1992; Stearns 1992; Skorping and Read 1998). However, Lynch et al. (2008) used mathematical models to demonstrate that interventions that affect mortality rates of mature parasitic nematodes could have complicated effects on optimal age to maturity, regardless of whether mortality is size‐dependent or independent. They argued that where an intervention measure is continuously applied, the optimum age at maturity may be longer relative to a situation with no intervention and that parasites should benefit from a greater reproductive life span. Field experiments studying the evolutionary effects of anthelmintics on *Teladorsagia circumcincta* showed that worm size was consistently larger in resistant isolates when compared to susceptible isolates (Leignel and Cabaret 2001). Worryingly, if drug selection favors increased size at maturity then resistant worms may be more fecund than susceptibles. It would be interesting to measure size at maturity as well as other life‐history traits of our evolved lines and investigate whether any responses in such traits correlate with apparently reduced susceptibility to Ivermectin.

## Conclusions

Our inclusion of a novel treatment that controls for both the increased risk of mortality and changes in population size of drug‐treated populations raises the question of whether previous studies that have not incorporated such controls should be re‐evaluated. For example, Lopes et al. (2008) report the rapid evolution of resistance to Levamisole within 10 generations of exposure under very similar experimental conditions to this study. Levamisole was administered at a concentration lethal for 75% of the ancestral population. A resistance bioassay was then performed on samples from generations 10 and 20, which showed a 25% increase in survival of populations under drug selection at generations 10 and 20. However, as there was no control for mortality between drug‐treated and control populations, it is difficult to assess whether there were effects of differences in density and mortality between treatments. We recommend that future work on resistance should incorporate adequate controls for parasite/pest density when assessing drug resistance evolution. In addition, controlling for differences in population size and rate of mortality could be implemented in any experimental evolution study where the selective agent induces greater mortality than control treatments.

Standing genetic variation in the form of susceptibility to chemical applications is important in the study of resistance evolution (Gilleard and Beech 2007). This study suggests there may be a complex relationship between the intensity of selection and, density‐dependent regulatory processes and life history of populations challenged with control measures. How these factors interact and affect characteristics such as tolerance and resistance could result in significant impacts on the evolution of susceptibility. For instance, studies of drug susceptibility in nematodes have shown that environments where conditions are inhospitable to free‐living larvae, which reduces larval densities, promote the evolution of resistance (Besier and Love 2003; Lawrence et al. 2007; Leathwick and Besier 2014). What proportion of this reported resistance is due to drug application or tolerance, and how it interacts with life history, is difficult to establish in the field. In order to understand how drug tolerance and resistance evolution may interact, future research should aim to identify precisely which traits are associated with tolerance and what influence they may have on resistance. The *Caenorhabditis* system allows a range of traits to be assessed over the course of selection experiments (Gray and Cutter 2014), and therefore should provide an invaluable model to explore factors which may affect the evolution of resistance and tolerance.

## Data archiving statement

Data available from the Dryad Digital Repository: http://dx.doi:10.5061/dryad.734c8.

## Supporting information


**Figure S1.** Relationship between survival and dose of Ivermectin for the SP8 strain of *Caenorhabditis remanei*.

**Figure S2.** Larval density over the course of the original selection experiment.

**Figure S3.** Seventy‐five hour survival of high dose, high random and zero dose lines when exposed to the high dose of Ivermectin used during selection.

**Figure S4.** Fifty‐two hour survival when exposed to the three drug doses used during selection (A = high; B = low: C and D = zero) of samples taken from generations 0, 5 and 10 during selection.

**Table S1.** Effect of treatment during selection (mortality treatment) on survivorship (Surv.diff) at two different time points (Bioassay), in drug‐treated environments (dose); assessed by null models where survival is constrained to be equal across treatments (see Data S1), using likelihood ratio tests of best the fitting model.
**Table S2.** Predicted theoretical loss of genetic diversity based on a simple population genetic model during the course of selection (generation) in HR (high random) and Z (zero dose) lines.
**Data S1.** Example R code for bioassay data analysis.

**Data S2.** Supplementary material: statistical methods and drift and loss of diversity.

## References

[eva12376-bib-0001] Allendorf, F. W. 1986 Genetic drift and the loss of alleles versus heterozygosity. Zoo Biology 5:181–190.

[eva12376-bib-0002] Ardelli, B. F. , L. E. Stitt , J. B. Tompkins , and R. K. Prichard 2009 A comparison of the effects of ivermectin and moxidectin on the nematode *Caenorhabditis elegans* . Veterinary Parasitology 165:96–108. 19631471 10.1016/j.vetpar.2009.06.043

[eva12376-bib-0003] Barnes, E. H. , R. J. Dobson , and I. A. Barger 1995 Worm control and anthelmintic resistance: adventures with a model. Parasitology Today 11:56–63. 15275374 10.1016/0169-4758(95)80117-0

[eva12376-bib-0004] Barrière, A. , and M.‐A. Félix 2007 Temporal dynamics and linkage disequilibrium in natural *Caenorhabditis elegans* populations. Genetics 176:999–1011. 17409084 10.1534/genetics.106.067223 PMC1894625

[eva12376-bib-0005] Barros, A. T. , J. Ottea , D. Sanson , and L. D. Foil 2001 Horn fly (Diptera: Muscidae) resistance to organophosphate insecticides. Veterinary Parasitology 96:243–256. 11240098 10.1016/s0304-4017(00)00435-0

[eva12376-bib-0006] Bates, D. , M. Maechler , B. Bolker , and S. Walker 2014 lme4: Linear mixed‐effects models using Eigen and S4. R Package Version 1.1‐7. http://cran.r-project.org/package=lme4 (accessed on 10 January 2016).

[eva12376-bib-0007] Besier, R. B. , and S. C. J. Love 2003 Anthelmintic resistance in sheep nematodes in Australia: the need for new approaches. Australian Journal of Experimental Agriculture 43:1383.

[eva12376-bib-0008] Brausch, J. M. , and P. N. Smith 2009 Development of resistance to cyfluthrin and naphthalene among *Daphnia magna* . Ecotoxicology 18:600–609. 19399609 10.1007/s10646-009-0318-1

[eva12376-bib-0009] Brenner, S. 1974 The genetics of *Caenorhabditis elegans* . Genetics 77:71–94. 4366476 10.1093/genetics/77.1.71 PMC1213120

[eva12376-bib-0010] Browne, W. J. , S. V. Subramanian , K. Jones , and H. Goldstein 2005 Variance partitioning in multilevel logistic models that exhibit overdispersion. Journal of the Royal Statistical Society: Series A (Statistics in Society) 168:599–613.

[eva12376-bib-0011] Busi, R. , and S. B. Powles 2009 Evolution of glyphosate resistance in a lolium rigidum population by glyphosate selection at sublethal doses. Heredity 103:318–325. 19491925 10.1038/hdy.2009.64

[eva12376-bib-0012] Carriere, Y. , J.‐P. Deland , D. A. Roff , and C. Vincent 1994 Life‐history costs associated with the evolution of insecticide resistance. Proceedings of the Royal Society of London B: Biological Sciences 258:35–40.

[eva12376-bib-0013] Chehresa, A. , R. N. Beech , and M. E. Scott 1997 Life‐history variation among lines isolated from a laboratory population of *Heligmosomoides polygyrus bakeri* . International Journal for Parasitology 27:541–551. 9193948 10.1016/s0020-7519(97)00005-2

[eva12376-bib-0014] Churcher, T. S. , and M.‐G. Basáñez 2008 Density dependence and the spread of anthelmintic resistance. Evolution 62:528–537. 17983465 10.1111/j.1558-5646.2007.00290.x

[eva12376-bib-0015] Churcher, T. S. , J.‐N. Filipe , and M.‐G. Basáñez 2006 Density dependence and the control of helminth parasites. Journal of Animal Ecology 75:1313–1320. 17032363 10.1111/j.1365-2656.2006.01154.x

[eva12376-bib-0016] Coles, T. B. , and M. W. Dryden 2014 Insecticide/acaricide resistance in fleas and ticks infesting dogs and cats. Parasites & Vectors 7:8. 24393426 10.1186/1756-3305-7-8 PMC3891977

[eva12376-bib-0017] Coles, G. C. , A. C. Rhodes , and A. J. Wolstenholme 2005 Rapid selection for ivermectin resistance in *Haemonchus contortus* . Veterinary Parasitology 129:345–347. 15845291 10.1016/j.vetpar.2005.02.002

[eva12376-bib-0018] Committee on Strategies for the Management of Pesticide Resistant Pest Populations, National Research Council 1986 Pesticide Resistance: Strategies and Tactics for Management. The National Academies Press, Washington, DC.

[eva12376-bib-0019] Cutter, A. D. , S. E. Baird , and D. Charlesworth 2006 High nucleotide polymorphism and rapid decay of linkage disequilibrium in wild populations of *Caenorhabditis remanei* . Genetics 174:901–913. 16951062 10.1534/genetics.106.061879 PMC1602088

[eva12376-bib-0020] Dey, S. , J. Bose , and A. Joshi 2012 Adaptation to larval crowding in *Drosophila ananassae* leads to the evolution of population stability. Ecology and Evolution 2:941–951. 22837839 10.1002/ece3.227 PMC3399160

[eva12376-bib-0021] Diaz, S. A. , J. Lindström , and D. T. Haydon 2008 Basic demography of *Caenorhabditis remanei* cultured under standard laboratory conditions. Journal of Nematology 40:167–178. 19440256 PMC2664667

[eva12376-bib-0022] Driscoll, M. 1989 Genetic and molecular analysis of a *Caenorhabditis elegans* beta‐tubulin that conveys benzimidazole sensitivity. The Journal of Cell Biology 109:2993–3003. 2592410 10.1083/jcb.109.6.2993 PMC2115974

[eva12376-bib-0023] Fritzsche, K. , N. Timmermeyer , M. Wolter , and N. K. Michiels 2014 Female, but not male, nematodes evolve under experimental sexual coevolution. Proceedings of the Royal Society of London B: Biological Sciences 281:20140942. 10.1098/rspb.2014.0942 PMC4213631 25339719

[eva12376-bib-0024] Fuller, R. C. , C. F. Baer , and J. Travis 2005 How and when selection experiments might actually be useful. Integrative and Comparative Biology 45:391–404. 21676785 10.1093/icb/45.3.391

[eva12376-bib-0025] Galvani, A. , and S. Gupta 1998 The effects of mating probability on the population genetics of nematodes. Journal of Helminthology 72:295. 9858624 10.1017/s0022149x00016631

[eva12376-bib-0027] Gassmann, A. J. , Y. Carrière , and B. E. Tabashnik 2009 Fitness costs of insect resistance to *Bacillus thuringiensis* . Annual Review of Entomology 54:147–163. 10.1146/annurev.ento.54.110807.090518 19067630

[eva12376-bib-0028] Ghosh, R. , E. C. Andersen , J. Shapiro , J. P. Gerke , and L. Kruglyak 2012 Natural variation in a chloride channel subunit confers avermectin resistance in *C. elegans* . Science 335:574–578. 22301316 10.1126/science.1214318 PMC3273849

[eva12376-bib-0029] Gilleard, J. S. , and R. N. Beech 2007 Population genetics of anthelmintic resistance in parasitic nematodes. Parasitology 134(Pt 8):1133–1147. 17608973 10.1017/S0031182007000066

[eva12376-bib-0030] Gray, J. C. , and A. D. Cutter 2014 Mainstreaming *Caenorhabditis elegans* in experimental evolution. Proceedings of the Royal Society of London B: Biological Sciences 281:20133055. 10.1098/rspb.2013.3055 PMC3906948 24430852

[eva12376-bib-0031] Greene, B. Y. , E. Shannon , M. A. Riley , and A. Read 2012 Moving Targets : Fighting the Evolution of Resistance in Infections, Pests, and Cancer. The American Academy of Microbiology Colloquim, Philadelphia, PA. 32865931

[eva12376-bib-0032] Hendry, A. P. , M. T. Kinnison , M. Heino , T. Day , T. B. Smith , G. Fitt , C. T. Bergstrom et al. 2011 Evolutionary principles and their practical application. Evolutionary Applications 4:159–183. 25567966 10.1111/j.1752-4571.2010.00165.x PMC3352551

[eva12376-bib-0033] Hope, I. A. 2001 C. elegans: A Practical Approach. Oxford University Press, Oxford.

[eva12376-bib-0034] Jackson, F. 1993 Anthelmintic resistance – the state of play. The British Veterinary Journal 149:123–138. 8485639 10.1016/S0007-1935(05)80083-1

[eva12376-bib-0035] James, C. E. , and M. W. Davey 2009 Increased expression of ABC transport proteins is associated with ivermectin resistance in the model nematode *Caenorhabditis elegans* . International Journal for Parasitology 39:213–220. 18708066 10.1016/j.ijpara.2008.06.009

[eva12376-bib-0036] James, C. E. , A. L. Hudson , and M. W. Davey 2009 Drug resistance mechanisms in helminths: is it survival of the fittest? Trends in Parasitology 25:328–335. 19541539 10.1016/j.pt.2009.04.004

[eva12376-bib-0037] Jansen, M. , A. Coors , R. Stoks , and L. De Meester 2011 Evolutionary ecotoxicology of pesticide resistance: a case study in *Daphnia* . Ecotoxicology 20:543–551. 21380529 10.1007/s10646-011-0627-z

[eva12376-bib-0038] Johnson, C. D. , S. J. E. Barry , H. M. Ferguson , and P. Müller 2015 Power analysis for generalized linear mixed models in ecology and evolution. Edited by Holger Schielzeth. Methods in Ecology and Evolution 6:133–142. 25893088 10.1111/2041-210X.12306 PMC4394709

[eva12376-bib-0039] Joshi, A. , N. G. Prasad , and M. Shakarad 2001 K‐selection, alpha‐selection, effectiveness, and tolerance in competition: density‐dependent selection revisited. Journal of Genetics 80:63–75. 11910126 10.1007/BF02728332

[eva12376-bib-0040] Kaplan, R. M. 2004 Drug resistance in nematodes of veterinary importance: a status report. Trends in Parasitology 20:477–481. 15363441 10.1016/j.pt.2004.08.001

[eva12376-bib-0041] Kaplan, R. M. , and A. N. Vidyashankar 2011 An inconvenient truth: global worming and anthelmintic resistance. Veterinary Parasitology 186:70–78. 22154968 10.1016/j.vetpar.2011.11.048

[eva12376-bib-0042] Kliot, A. , and M. Ghanim 2012 Fitness costs associated with insecticide resistance. Pest Management Science 68:1431–1437. 22945853 10.1002/ps.3395

[eva12376-bib-0043] Lawrence, K. E. , D. M. Leathwick , A. P. Rhodes , R. Jackson , C. Heuer , W. E. Pomroy , D. M. West et al. 2007 Management of gastrointestinal nematode parasites on sheep farms in New Zealand. New Zealand Veterinary Journal 55:228–234. 17928899 10.1080/00480169.2007.36773

[eva12376-bib-0044] Leathwick, D. M. , and R. B. Besier 2014 The management of anthelmintic resistance in grazing ruminants in Australasia–strategies and experiences. Veterinary Parasitology 204:44–54. 24439840 10.1016/j.vetpar.2013.12.022

[eva12376-bib-0045] Leathwick, D. M. , B. C. Hosking , S. A. Bisset , and C. H. McKay 2009 Managing anthelmintic resistance: is it feasible in New Zealand to delay the emergence of resistance to a new anthelmintic class? New Zealand Veterinary Journal 57:181–192. 19649011 10.1080/00480169.2009.36900

[eva12376-bib-0046] Leignel, V. , and J. Cabaret 2001 Massive use of chemotherapy influences life traits of parasitic nematodes in domestic ruminants. Functional Ecology 15:569–574.

[eva12376-bib-0047] Lopes, P. C. , E. Sucena , M. E. Santos , and S. Magalhães 2008 Rapid experimental evolution of pesticide resistance in *C. elegans* entails no costs and affects the mating system. PLoS ONE 3:e3741. 19011681 10.1371/journal.pone.0003741 PMC2580027

[eva12376-bib-0048] Lynch, P. A. , U. Grimm , and A. F. Read 2008 How will public and animal health interventions drive life‐history evolution in parasitic nematodes? Parasitology 135:1599–1611. 18328116 10.1017/S0031182008000309

[eva12376-bib-0049] Manalil, S. , R. Busi , M. Renton , and S. B. Powles 2011 Rapid evolution of herbicide resistance by low herbicide dosages. Weed Science 59:210–217.

[eva12376-bib-0050] Morand, S. 1996 Life‐history traits in parasitic nematodes: a comparative approach for the search of invariants. Functional Ecology 10:210–218.

[eva12376-bib-0051] Morran, L. T. , O. G. Schmidt , I. A. Gelarden , R. C. Parrish , and C. M. Lively 2011 Running with the red queen: host‐parasite coevolution selects for biparental sex. Science 333:216–218. 21737739 10.1126/science.1206360 PMC3402160

[eva12376-bib-0052] Mueller, L. D. 1988 Evolution of competitive ability in *Drosophila* by density‐dependent natural selection. Proceedings of the National Academy of Sciences of the USA 85:4383–4386. 3132712 10.1073/pnas.85.12.4383 PMC280433

[eva12376-bib-0053] Palumbi, S. R. , and P. Mu 2001 Humans as the world's greatest evolutionary force the pace of human‐induced evolution. Science 293:1786–1790. 11546863 10.1126/science.293.5536.1786

[eva12376-bib-0054] Prasad, N. G. , and A. Joshi 2003 What have two decades of laboratory life‐history evolution studies on *Drosophila melanogaster* taught us? Journal of Genetics 82:45–76. 14631102 10.1007/BF02715881

[eva12376-bib-0055] Puniamoorthy, N. , M. A. Schäfer , J. Römbke , R. Meier , and W. U. Blanckenhorn 2014 Ivermectin sensitivity is an ancient trait affecting all ecdysozoa but shows phylogenetic clustering among sepsid flies. Evolutionary Applications 7:548–554. 24944568 10.1111/eva.12152 PMC4055176

[eva12376-bib-0056] R Core Team 2014 R: A Language and Environment for Statistical Computing. R Foundation for Statistical Computing, Vienna.

[eva12376-bib-0057] Ranjan, S. , G. T. Wang , C. Hirschlein , and K. L. Simkins 2002 Selection for resistance to macrocyclic lactones by *Haemonchus contortus* in sheep. Veterinary Parasitology 103:109–117. 11751006 10.1016/s0304-4017(01)00551-9

[eva12376-bib-0058] REX Consortium 2010 The skill and style to model the evolution of resistance to pesticides and drugs. Evolutionary Applications 3:375–390. 25567932 10.1111/j.1752-4571.2010.00124.x PMC3352466

[eva12376-bib-0059] REX Consortium 2013 Heterogeneity of selection and the evolution of resistance. Trends in Ecology & Evolution 28:110–118. 23040463 10.1016/j.tree.2012.09.001

[eva12376-bib-0060] Ritz, C. , and J. C. Streibig 2007 Bioassay analysis using R. Journal of Statistical Software 12:1–18.

[eva12376-bib-0061] Roff, D. A. 1992 The Evolution of Life Histories: Theory and Analysis. Chapman & Hall, New York, NY.

[eva12376-bib-0062] Roush, R. T. , and J. A. McKenzie 1987 Ecological genetics of insecticide and acaricide resistance. Annual Review of Entomology 32:361–380. 10.1146/annurev.en.32.010187.002045 3545056

[eva12376-bib-0100] Rufener, L. , J. Keiser , R. Kaminsky , P. Mäser , and D. Nilsson 2010 Phylogenomics of Ligand‐Gated Ion Channels Predicts Monepantel Effect. PLoS Pathogens 6:e1001091. 20838602 10.1371/journal.ppat.1001091 PMC2936538

[eva12376-bib-0063] Sangster, N. C. , and J. Gill 1999 Pharmacology of anthelmintic resistance. Parasitology Today 15:141–146. 10322335 10.1016/s0169-4758(99)01413-1

[eva12376-bib-0064] Scott, J. A. 1995 The molecular genetics of resistance: resistance as a response to stress. Symposium on Pesticide Resistance 78:399–414.

[eva12376-bib-0065] Shi, M. , P. J. Collins , T. J. Ridsdill‐Smith , R. N. Emery , and M. Renton 2013 Dosage consistency is the key factor in avoiding evolution of resistance to phosphine and population increase in stored‐grain pests. Pest Management Science 69:1049–1060. 23292953 10.1002/ps.3457

[eva12376-bib-0066] Simpkin, K. G. , and G. C. Coles 1981 The use of *Caenorhabditis elegans* for anthelmintic screening. Journal of Chemical Technology and Biotechnology 31:66–69.

[eva12376-bib-0067] Skorping, A. , and A. F. Read 1998 Drugs and parasites: global experiments in life history evolution? Ecology Letters 1:10–12.

[eva12376-bib-0068] Sparks, T. C. , J. E. Dripps , G. B. Watson , and D. Paroonagian 2012 Resistance and cross‐resistance to the spinosyns – a review and analysis. Pesticide Biochemistry and Physiology 102:1–10.

[eva12376-bib-0069] Stearns, S. C. 1992 The Evolution of Life Histories. Oxford University Press, Oxford, UK.

[eva12376-bib-0070] Tabashnik, B. E. , D. Mota‐Sanchez , M. E. Whalon , R. M. Hollingworth , and Y. Carrière 2014 Defining terms for proactive management of resistance to Bt crops and pesticides. Journal of Economic Entomology 107:496–507. 24772527 10.1603/ec13458

[eva12376-bib-0071] Taylor, C. E. , F. Quaglia , and G. P. Georghiou 1983 Evolution of resistance to insecticides: a cage study on the influence of migration and insecticide decay rates!. Journal of Economic Entomology 76:704–707.

[eva12376-bib-0072] Wolstenholme, A. J. , I. Fairweather , R. Prichard , G. von Samson‐Himmelstjerna , and N. C. Sangster 2004 Drug resistance in veterinary helminths. Trends in Parasitology 20:469–476. 15363440 10.1016/j.pt.2004.07.010

