# Supplementary figures and images for: Evolution of drug‐tolerant nematode populations in response to density reduction
Source: Evol Appl. 2016 Mar 29;9(5):726–38. doi: 10.1111/eva.12376 (PMC4869413; doi:10.1111/eva.12376)

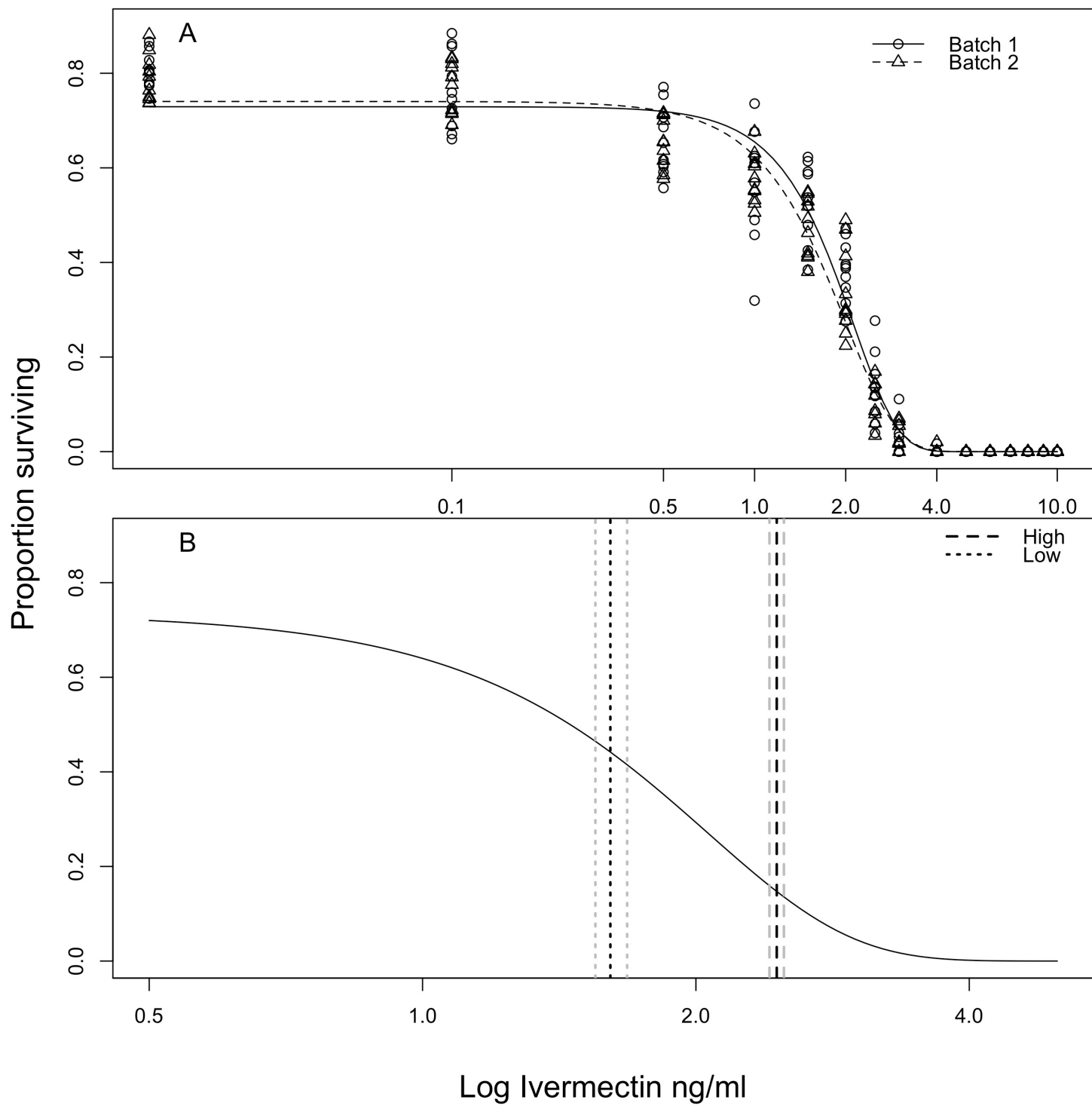

Supplement: Supplementary file 1 — Figure S1. Relationship between survival and dose of Ivermectin for the SP8 strain of Caenorhabditis remanei. [file EVA-9-726-s001.pdf]

Number of juveniles

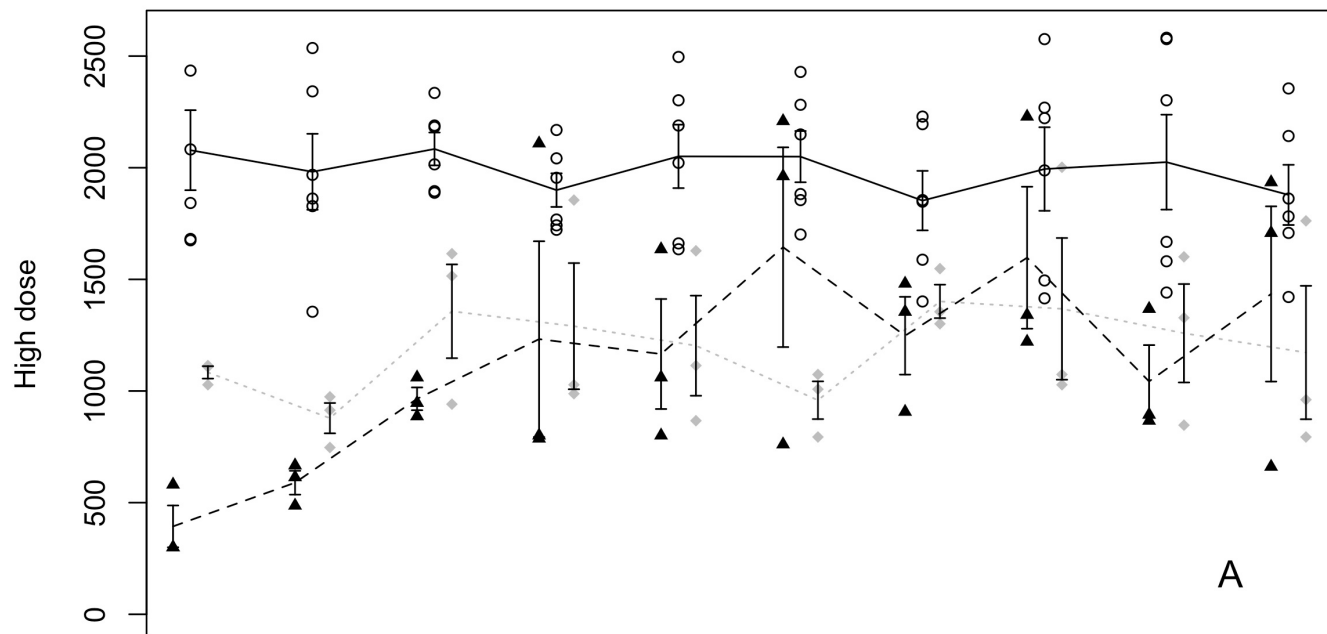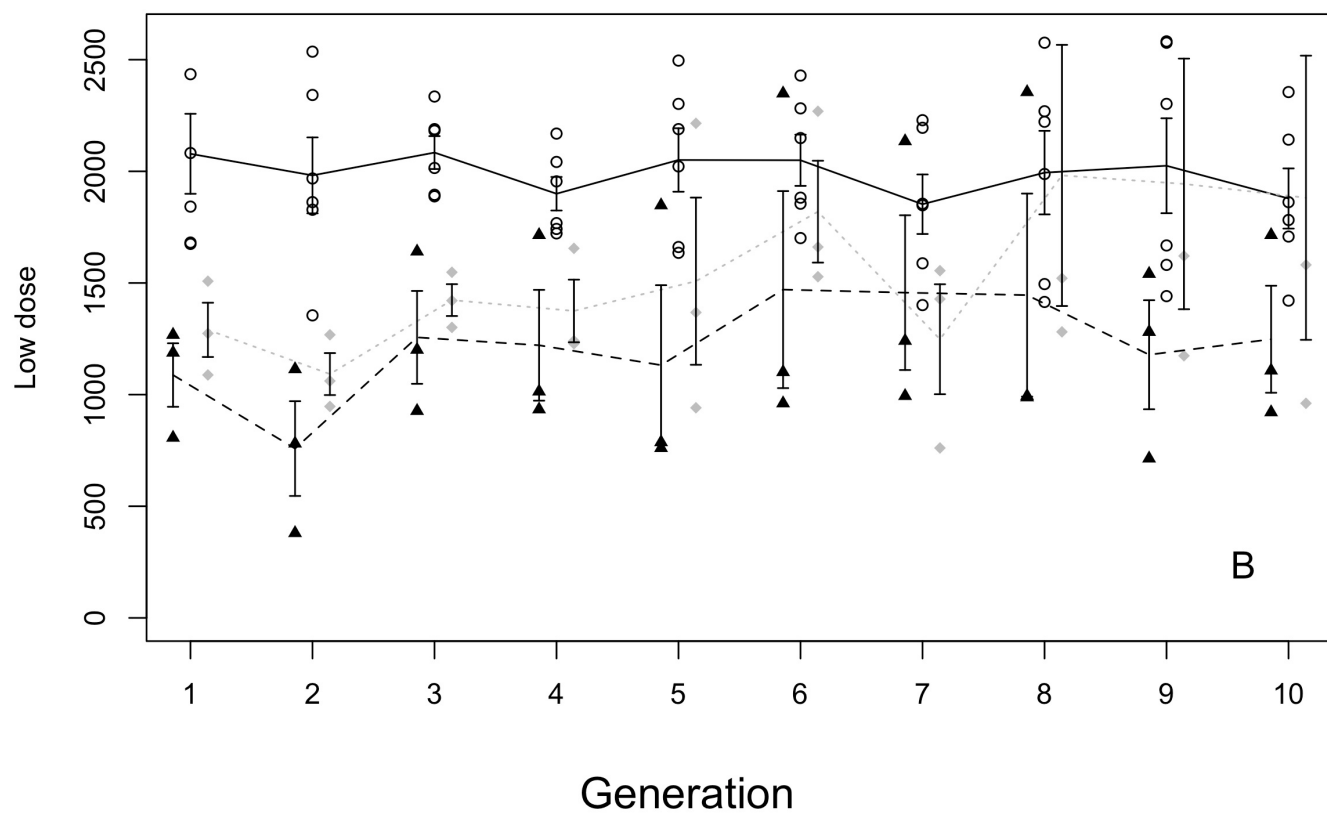

Supplement: Supplementary file 2 — Figure S2. Larval density over the course of the original selection experiment. [file EVA-9-726-s002.pdf]

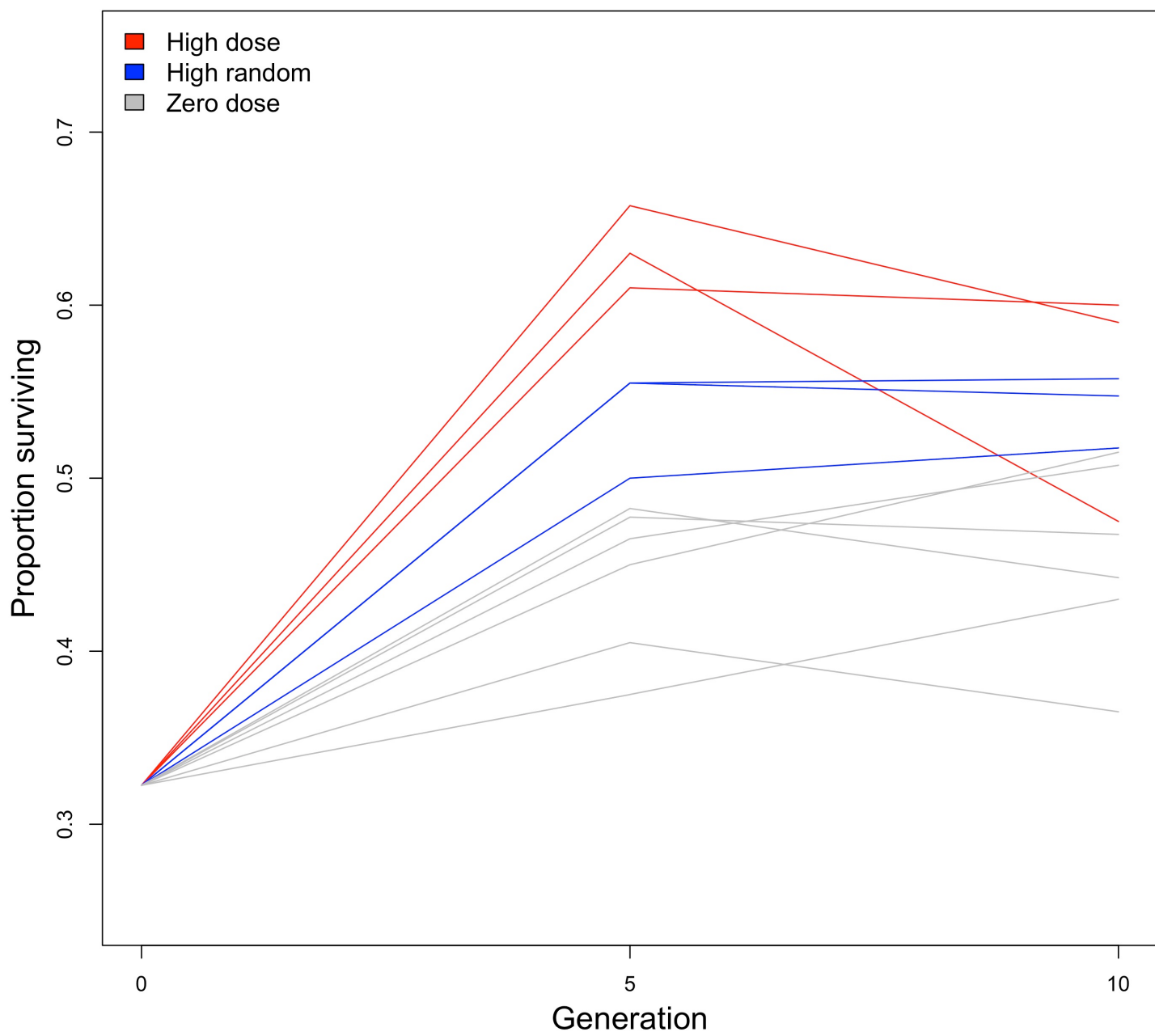

Supplement: Supplementary file 3 — Figure S3. Seventy‐five hour survival of high dose, high random and zero dose lines when exposed to the high dose of Ivermectin used during selection. [file EVA-9-726-s003.pdf]

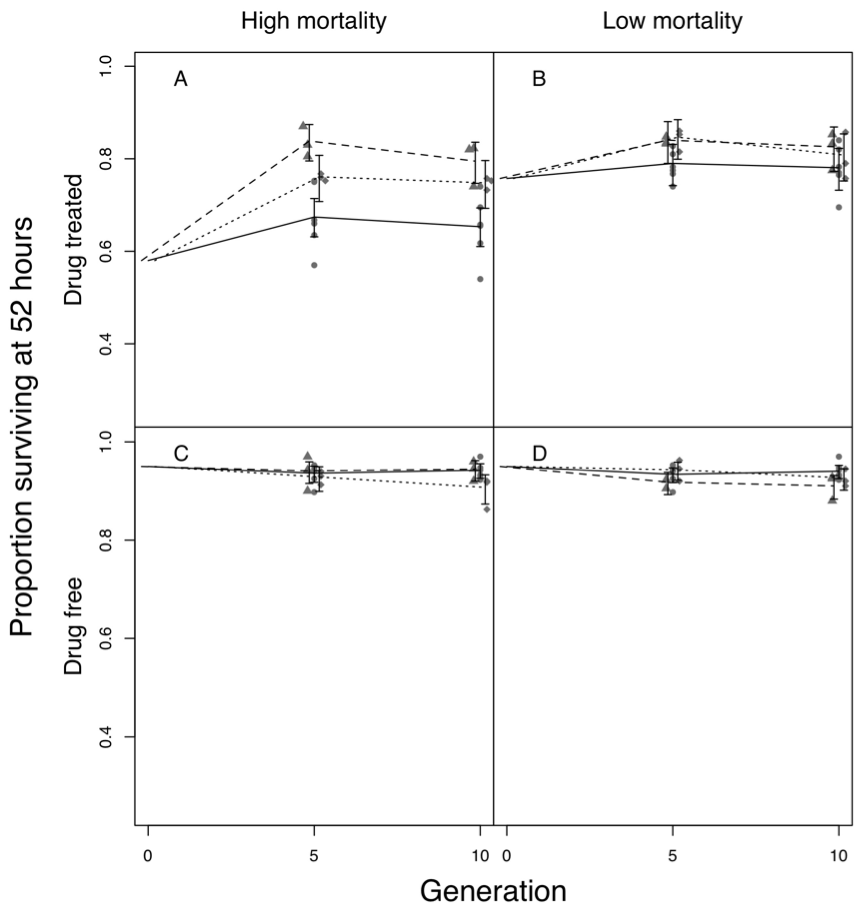

Supplement: Supplementary file 4 — Figure S4. Fifty‐two hour survival when exposed to the three drug doses used during selection (A = high; B = low: C and D = zero) of samples taken from generations 0, 5 and 10 during selection. [file EVA-9-726-s004.tiff]
