# Supplementary material for: Evolution of drug‐tolerant nematode populations in response to density reduction
Source: Evol Appl. 2016 Mar 29;9(5):726–38. doi: 10.1111/eva.12376 (PMC4869413; doi:10.1111/eva.12376)
Supplement: Supplementary file 5 — Table S1. Effect of treatment during selection (mortality treatment) on survivorship (Surv.diff) at two different time points (Bioassay), in drug‐treated environments (dose); assessed by null models where survival is constrained to be equal across treatments (see Data S1), using likelihood ratio tests of best the fitting model. Table S2. Predicted theoretical loss of genetic diversity based on a simple population genetic model during the course of selection (generation) in HR (high random) and Z (zero dose) lines. Data S1. Example R code for bioassay data analysis. [file EVA-9-726-s005.docx]

**Supplementary material**

*Statistical methods*

All statistical analyses were performed using R v 3.1.2 (R Core Team 2014) and we defined a significance threshold of P = 0.05 for all tests. Our first question was concerned with drug dose efficacy and asked ‘What is the relationship between *C. remanei* survival and Ivermectin dose over a range of concentrations?’ The doses required to cause 40% and 80% mortality were estimated, with 95% CI’s, using the drc package (Ritz and Streibig 2007). In order to calculate estimates of these two doses we constructed a dose response curve of the relationship between worm survival and concentration of Ivermectin. Dose response curves are generally sigmoidal and can be defined by four parameters: the first two parameters are the upper and lower asymptote, where changes in concentration have no effect on survival (Ritz 2010). The third parameter is the slope of the curve, which defines the potency of the drug where efficacy increases from zero to its maximum, the more potent a drug is the steeper the curve will be. The fourth parameter is the inflection point, where the curve changes from concave to convex and is located at or near the LD50 (dose causing 50% mortality) for the drug. We fitted a range of dose-response models (log-logistic, Weibull-1 and Weibull-2) with the lower asymptote fixed at 0% survival and used maximum likelihood to select the most appropriate model of survival data. Ivermectin concentration and batch were fitted as fixed effects in our full model. To assess whether the relationship between survivorship and Ivermectin concentration remained the same between batches performed at different times (i.e. repeatability), batch was removed from the model and tested against the full model. Estimates of the required doses, with 95% CIs, were then derived from model predictions.

Our three remaining questions involved potential changes in survival of evolved lines used in the selection experiment, which could result in differential survival between treatments. We addressed these questions with a common general approach described below. We conducted the analyses on discrete data sets from the resistance bioassays, which specifically addressed our research questions. In order to simplify our analyses, survival of high mortality (HD and HR) and low mortality treatments (LD and LR) were contrasted with control Z lines in separate tests, unless explicitly stated otherwise. The analysis compared survival of lines from the original selection experiment treatment groups (drug-treated, random mortality and zero dose) using generalised linear mixed models with the glmer function in the lme4 package and assuming a binomial error distribution with a logit link function (Bates et al 2014). Treatment and generation, as well as the interaction between them, were fitted as fixed effects. The evolutionary replicate (line) was fitted as a random effect. An observation-level random effect was fitted to account for any overdispersion between replicate lines in the selection experiment and repeated sampling of populations in the drug resistance bioassay (Browne et al. 2005). We refer to this model for the fixed effects as the full model. Treatment effects in the selection experiment were tested using likelihood ratio tests. The null hypothesis of no difference in survival between the three treatments (H_0_: Drug treatment = Random mortality = Zero dose) was tested by comparing the full model with a null model with no fixed effect of treatment or treatment x generation interaction. Generation was kept in the null model to account for any drift in survivorship. This first step of the analysis tested for significance of fixed effects and interactions between them. A further three post-hoc measures were then used to assess the effects of individual treatments; differences in survival in the drug treatment relative to the zero dose treatment were tested by comparing the full model to a null model where survival in drug and zero dose treatments were constrained to be equal (H_0_: Drug = Zero). Differential survival in response to the random mortality treatment (H_0_: Random = Zero) and density-dependence in response to selection (H_0_: Drug treatment = Random treatment) were tested in the same way. This general approach was used throughout the analyses to answer our research questions by establishing any evidence of differential survival between treatments challenged with different drug doses and at different life-history stages.

Firstly we asked: ‘Is there an increase in survivorship across generations of populations selected in drug-treated environments, and does this vary with dosage?’ The formal assessment of whether heritable increases in survivorship occurred in drug-selected lines was tested by challenging drug-selected and zero dose lines to the same high and low doses of Ivermectin used during selection. Survival data of HD lines exposed to a high dose of Ivermectin was assessed by the null hypothesis of no difference in survival between the three treatments (H_0_: HD = HR = Z) using the general approach discussed. After establishing a significant effect of treatment on survival a post-hoc test was performed with the null hypothesis of no difference in survivorship between HD and Z lines (H_0_: HD = Z). Any evolved increase in the survivorship of LD lines relative to Z lines was assessed under the null hypothesis (H_0_: LD = LR = Z) and if treatment was significant, a post-hoc test was applied to assess differential survival between drug-treated and control lines (H_0_: LD = Z).

Secondly we asked ‘Does density-dependent selection affect the apparent evolution of resistance in selected lines?’ We challenged random mortality (HR and LR) treated lines to both high and low doses of Ivermectin and again used our general approach to assess differential survival between selection experiment treatments. Differences in survivorship between high mortality treatments where the null model (H_0_: HD = HR = Z) was rejected were tested with two post-hoc tests. The first tested for a difference in survival between HR and Z lines, and the second, differential survival between HD and Z lines. Assessment of low mortality lines was conducted using exactly the same approach but with the null model (H_0_: LD = LR = Z).

Thirdly we asked ‘Is there a cost of adaptation to drug-treated environments in terms of survival in drug-free environments?’ We answered this question by exposing evolved lines to a drug-free environment and used our general approach to assess differences in survival between treatments, with the assumption that if there were a cost to adaptation then drug-treated lines would show lower survival than control (Z) lines.

Finally, we asked ‘Does survival of different life-history stages (juvenile and adult) respond to drug-selection in the same way? To answer this question we conducted the analyses used in questions two to four separately on survival data collected at 52 and 75 hours and informally compared differences of model predictions from the two time points. In addition, we pooled our whole datasets from the resistance bioassays using survival data from all drug-treated environments at both 52 hours (juvenile survival) and 75 hours (adult survival) and asked whether there were any interactions between selection experiment treatment, bioassay dosage and life-history stage. This was done for generations 5 and 10 independently to account for any changes in survival over generations. If there was no evidence of a three-way interaction we explored two-way interactions. We assumed that any evidence of interactions of life-history stage with treatment, dosage or both suggests that survival responded differently in juveniles and adults. We used a full model with fixed effects of treatment, bioassay dosage and life-history stage, as well as all possible two-way interactions between them. Random effects were: selection line, bioassay replicate and an observation-level random intercept. The full model was compared with a null model without the interaction of interest using a likelihood ratio test to assess the significance of interactions.

*Drift and loss of genetic diversity*

The evolutionary potential of small populations may be affected by drift due to the loss of genetic variation (Allendorf 1986). During the course of the selection experiment, population sizes varied both between lines within a treatment, and between treatments, due to bottlenecks imposed by drug application or random mortality. These bottlenecks could have resulted in a reduction in genetic diversity. Although we have no data on levels of genetic diversity it is worth considering the risk and extent of the loss of genetic variation in the selected lines used in this study. Allendorf (1986) outlines three useful measures of genetic variation and its loss: 1) changes in heterozygosity; 2) loss of multiple equally frequent alleles and 3) loss of rare alleles. Using census data from the selection experiment each measure of genetic variation was estimated for the HR and zero dose treatments at each generation (Table S2). Predicted changes in heterozygosity were calculated using equation (1) where *N* is population size. The expected proportion of the original heterozygosity remaining after each generation is:

$$1- \frac{1}{2N} (1)$$

This expectation is only valid if there are no selective differences between alleles; all other genetic diversity calculations in this paper also make this assumption of allelic neutrality. Predicted changes in the loss of multiple equally frequent alleles were calculated using equation (2).

$$E\left( n^{'} \right)= n- \sum_{j=1}^{n} (1- p_{j})^{2N} (2)$$

Where *n* equals the number of alleles in the population, and *p* the frequency of an allele. We calculated the predicted number of alleles present at each generation of the selection experiment assuming the initial number of alleles for a given locus was 10. This potentially overestimates genetic diversity within our study species but *C. remanei* have been shown to be a particularly genetically diverse species (Cutter, Baird, and Charlesworth 2006; Dey et al. 2013). The greater the numbers of alleles are at a given locus, the more likely it is an allele will be lost where alleles are considered to have equal frequency. Therefore, our high estimate of initial allele numbers may exaggerate the risk of allele loss; lower estimates of initial allele numbers would result in allele loss being less likely. Rare alleles (P < 0.01) are especially susceptible to loss during a bottleneck. The probability of losing a rare allele with frequency p = 0.01 is given by equation (3).

$$(1- p_{j})^{2N} (3)$$

An assumption was made that rare alleles were present at a frequency of 0.01, as this should provide some measure of the potential for rare allele loss in both HR and Z lines during the course of the selection experiment.

In HR lines, predicted heterozygosity decreased by 10% and 15% at generations 5 and 10, respectively (Table S2) whereas estimated heterozygosity of Z lines decreased by 5% and 10% (Table S2). The estimated number of alleles present in HR lines fell from 10 to 9.35 and 9.24 at generations 5 and 10, respectively, whereas in Z lines there was no decrease over 10 generations (Table S2). The high cumulative risk of loss of rare alleles by generation 5 (Table S2), suggests that any rare alleles would be unlikely to persist within both HR and Z lines. Heterozygosity and the number of alleles remaining after a bottleneck are expected to be important in terms of a population’s ability to respond to selection. Populations subjected to bottlenecks such as the HD and HR treatments in this study could be subject to considerable drift as a result of bottlenecking events. The increase in survivorship of High-dose and random-mortality lines could be a result of loss of genetic variation due to drift, and would require that all populations drifted in the same direction. However, our theoretical predictions of the loss of genetic diversity in HR and Z lines suggest that both treatments went through similar losses of genetic diversity. Predicted heterozygosity and the total number of alleles did decrease more rapidly in HR lines relative to Z lines but the difference between the two treatments was small. In the case of rare alleles, it is likely that any rare allele would have been lost from populations in both HR and Z lines. Thus, it seems reasonable that any evolved increase in survivorship of random-mortality and potentially drug-treated lines, was due to ecological processes occurring as a consequence of density-dependent selection and not loss of genetic variation due to drift.

*References*

Allendorf, F. W. 1986. “Genetic Drift and the Loss of Alleles versus Heterozygosity.” *Zoo Biology* 5 (2): 181–90. doi:10.1002/zoo.1430050212.

Bates D., M. Maechler, B. Bolker, and S. Walker. 2014. “lme4: Linear Mixed-Effects Models Using Eigen and S4. R Package Version 1.1-7.” http://cran.r-project.org/package=lme4.

Browne, W. J., S. V. Subramanian, K. Jones, and H. Goldstein. 2005. “Variance Partitioning in Multilevel Logistic Models That Exhibit Overdispersion.” *Journal of the Royal Statistical Society: Series A (Statistics in Society)* 168 (3): 599–613. doi:10.1111/j.1467-985X.2004.00365.x.

Cutter, A. D., S. E. Baird, and D. Charlesworth. 2006. “High Nucleotide Polymorphism and Rapid Decay of Linkage Disequilibrium in Wild Populations of Caenorhabditis Remanei.” *Genetics* 174 (2): 901–13. doi:10.1534/genetics.106.061879.

Dey, A., C. K. W. Chan, C. G. Thomas, and A. D. Cutter. 2013. “Molecular Hyperdiversity Defines Populations of the Nematode Caenorhabditis Brenneri.” *Proceedings of the National Academy of Sciences of the United States of America* 110 (27): 11056–60. doi:10.1073/pnas.1303057110.

R Core Team. 2014. “R: A Language and Environment for Statistical Computing.” Vienna: R Foundation for Statistical Computing.

Ritz, C. 2010. “Toward a Unified Approach to Dose-Response Modeling in Ecotoxicology.” *Environmental Toxicology and Chemistry / SETAC* 29 (1): 220–29. doi:10.1002/etc.7.

Ritz, C., and J. C. Streibig. 2007. “Bioassay Analysis Using R.” *Journal of Statistical Software* 12 (5): 1–18.

Table S1 Effect of treatment during selection (mortality treatment) on survivorship (Surv.diff) at two different time points (Bioassay), in drug-treated environments (dose); assessed by null models where survival is constrained to be equal across treatments (see Supplementary Methods), using likelihood ratio tests of best the fitting model. d.f.: Degrees of freedom. Surv.diff: Absolute difference in mean survival between the highlighted null model treatments (first minus second).

| Bioassay | Mortality treatment | Dose | Best fitting model | Null models | χ2 (d.f.) | P-value | Surv.diff | |
| --- | --- | --- | --- | --- | --- | --- | --- | --- |
|  |  |  |  |  |  |  | Gen 5 | Gen 10 |
| 52 hours | High | High | G + T | 1) HD = HR = Z | 16.21 (2) | 0.0003 |  |  |
|  |  |  |  | 2) HD = Z | 15.73 (1) | < 0.0001 | 0.16 | 0.14 |
|  |  |  |  | 3) HR = Z | 7.05 (1) | 0.0079 | 0.09 | 0.10 |
|  |  |  |  | 4) HD = HR | 4.18 (1) | 0.041 | 0.07 | 0.04 |
|  | Zero | G | 1) HD = HR = Z | 3.91 (2) | 0.14 |  |  |  |
|  | Low | Low | G + T | 1) LD = LR =Z | 6.71 (2) | 0.034 |  |  |
|  |  |  |  | 2) LD = Z | 5.21 (1) | 0.022 | 0.05 | 0.05 |
|  |  |  |  | 3) LR = Z | 4.43 (1) | 0.035 | 0.04 | 0.04 |
|  |  |  |  | 4) LD = LR | 0.06 (1) | 0.80 | 0.01 | 0.01 |
|  |  | Zero | G + T | 1) LD = LR = Z | 6.29 (2) | 0.043 |  |  |
|  |  |  |  | 2) LD = Z | 5.97 (1) | 0.015 | -0.02 | -0.02 |
|  |  |  |  | 3) LR = Z | 0.062 (1) | 0.80 | 0.00 | 0.00 |
|  |  |  |  | 4) LD = LR | 3.90 (1) | 0.14 | -0.02 | -0.02 |
| 75 hours | High | High | G + T + GxT | 1) HD = HR = Z | 22.26 (4) | 0.00018 |  |  |
|  |  |  |  | 2) HD = Z | 21.11 (2) | < 0.0001 | 0.19 | 0.10 |
|  |  |  |  | 3) HR = Z | 8.56 (2) | 0.014 | 0.09 | 0.09 |
|  |  |  |  | 4) HD = HR | 6.56 (2) | 0.038 | 0.10 | 0.01 |
|  |  | Zero | G | 1) HD = HR = Z | 3.59 (2) | 0.47 |  |  |
|  | Low | Low | G | 1) LD = LR = Z | 7.67 (2) | 0.11 |  |  |
|  |  | Zero | G + T + GxT | 1) LD = LR = Z | 11.47 (4) | 0.022 |  |  |
|  |  |  |  | 2) LD = Z | 11.33 (2) | 0.0035 | -0.01 | -0.06 |
|  |  |  |  | 3) LR = Z | 1.84 (2) | 0.40 | 0.00 | -0.02 |
|  |  |  |  | 4) LD = LR | 3.25 (2) | 0.20 | -0.01 | -0.04 |

G: Generation; T: Treatment; GxT: Generation x Treatment interaction; HD: High dose treatment; HR: High random treatment; LD: Low dose treatment; LR: Low random treatment; Z: Zero dose treatment.

Table S2 Predicted theoretical loss of genetic diversity based on a simple population genetic model during the course of selection (generation) in HR (High random) and Z (Zero dose) lines. Predictions of heterozygosity, the number of equally frequent alleles and risk of loss of rare alleles were made using mean population sizes for each treatment at each generation, using methods described by Allendorf (1986). Bold values show predicted diversity at generations 5 and 10, which correspond to when resistance bioassays were performed.

| Treatment | Generation | Population size | Heterozygosity* | Number of Alleles | Risk of loss of rare alleles (%) |
| --- | --- | --- | --- | --- | --- |
| HR | 1 | 15 | 0.97 | 9.58 | 74 |
| HR | 2 | 18 | 0.94 | 9.4 | 33 |
| HR | 3 | 28 | 0.92 | 9.38 | 18 |
| HR | 4 | 33 | 0.91 | 9.37 | 13 |
| HR | **5** | **33** | **0.9** | **9.35** | **13** |
| HR | 6 | 29 | 0.89 | 9.31 | 17 |
| HR | 7 | 35 | 0.88 | 9.29 | 12 |
| HR | 8 | 38 | 0.87 | 9.28 | 10 |
| HR | 9 | 31 | 0.86 | 9.25 | 15 |
| HR | **10** | **39** | **0.85** | **9.24** | **9** |
| Z | 1 | 47 | 0.99 | 10 | 39 |
| Z | 2 | 48 | 0.98 | 10 | 38 |
| Z | 3 | 49 | 0.97 | 10 | 37 |
| Z | 4 | 47 | 0.96 | 10 | 39 |
| Z | **5** | **46** | **0.95** | **10** | **40** |
| Z | 6 | 47 | 0.94 | 10 | 39 |
| Z | 7 | 47 | 0.93 | 10 | 39 |
| Z | 8 | 47 | 0.92 | 10 | 39 |
| Z | 9 | 47 | 0.91 | 10 | 39 |
| Z | **10** | **47** | **0.9** | **10** | **39** |

Number of alleles: an assumption of 10 alleles for a given locus was made; loss of rare alleles: rare alleles were assumed to at an initial frequency of 0.01. * Heterozygosity is the proportion of original heterozygosity remaining in the populations in each generation.

Fig S1 Relationship between survival and dose of Ivermectin for the SP8 strain of *C. remanei*. Panel A shows the relationship between survival and dose for two repeated assays (batches) accounting for differences in survival between dose-response assays performed on different dates. Points are individual replicates for each batch (triangles and circles). Panel B shows the doses used in the selection experiment (black broken lines) estimated to cause 40% and 80% mortality after taking into account any background mortality not due to the drug (LD40 and LD80). Grey broken lines show 95% confidence intervals around the estimated doses.

Fig S2 Larval density over the course of the original selection experiment. Lines represent mean number of juveniles for each treatment; points are the number of juveniles on day two of each generation for each replicate line within a treatment. Panel A shows density of high mortality lines: HD and HR with Z lines. Panel B shows density of low mortality lines: LD and LR with Z lines; circles, solid line = zero dose; triangles, dashed line = drug treatment; diamonds, dotted line = random mortality. Error bars; standard error for the mean number of juveniles.

Fig S3 Seventy-five hour survival of High dose, High random and Zero dose lines when exposed to the high dose of Ivermectin used during selection. Red, blue and grey lines show survival of replicate populations for each treatment during selection.

Fig S4 Fifty-two hour survival when exposed to the three drug doses used during selection (A = high; B = low: C and D = zero) of samples taken from generations 0, 5 and 10 during selection. Panels A and C show survivorship of high mortality lines: HD and HR. Panels B and D show survivorship of low mortality lines: LD and LR. Points are mean survival data for each replicate population, lines represent predictions of maximal models (generation + treatment + generation*treatment) for each treatment: circles, solid line = zero dose; triangles, dashed line = drug treatment; diamonds, dotted line = random mortality. Error bars; 95% confidence intervals for mean survival.
