# Supplementary material for: Evolution of drug‐tolerant nematode populations in response to density reduction
Source: Evol Appl. 2016 Mar 29;9(5):726–38. doi: 10.1111/eva.12376 (PMC4869413; doi:10.1111/eva.12376)
Supplement: Supplementary file 6 — Data S2. Supplementary material: statistical methods and drift and loss of diversity. [file EVA-9-726-s006.docx]

**Supplementary material**

*An example R code for running a GLMM on HD, HR and Z selected lines when exposed to a high dose of Ivermectin in the resistance bioassay*

# Adult (75 hour) survival data subset from whole bioassay dataset.

adult.rows <- bioassay$stage %in% c("adult")

adults <- bioassay[adult.rows,]

# Highdose resistance bioassay data subset from adult survival dataset.

highdose.rows <- adults$dose %in% c("highdose")

highdose <- adults[highdose.rows,]

# HD, RH and Z lines subset from highdose bioassay data used in GLMM.

which.rows <- highdose$Population %in% c("H1","H2","H3","RH1","RH2","RH3","Z1","Z2","Z3","Z4","Z5","Z6")

hd <- highdose[which.rows,]

# Call lme4

library(lme4)

# Define Treatment (Treat), generation (Gen), population and Replicate (Rep) as factors.

hd$fGen <- factor(hd$Gen)

hd$fRep <-factor(hd$Rep)

hd$fTreat <-factor(hd$Treat)

hd$fTreat <- as.character(hd$Treat)

hd$fTreat <- factor(hd$fTreat, c("Zero","RH","High"))

table(hd$fTreat)

hd$Population <- as.character(hd$Population)

hd$Population <- factor(hd$Population)

table(hd$Population,hd$Gen)

# Add an observation level intercept to account for overdispersion

hd$obs <- factor(1:nrow(hd))

# Full model used to assess differences in survival between treatments

fit1 <- glmer(cbind(alive, dead) ~ fGen + fTreat + fGen:fTreat + (1|Rep) + (1|Population) + (1|obs), family="binomial", data=hd)

summary(fit1)

Null.model <- glmer(cbind(alive, dead) ~ fGen + (1|Rep) + (1|Population) + (1|obs),

family="binomial", data=hd)

anova(fit1, Null.model)

# Breakdown treatment, generation and their interactions to allow contrasts to be made # between treatments

hd$Gen5 <- as.integer(hd$fGen=="5")

hd$Gen10 <- as.integer(hd$fGen=="10")

hd$RH <- as.integer(hd$fTreat=="RH")

hd$High <- as.integer(hd$fTreat=="High")

hd$Gen10xRH <- hd$Gen10*hd$RH

hd$Gen10xHigh <- hd$Gen10*hd$High

# Reconstruct “fit1” using terms for each level of treatment, generation and their interactions

fit2 <- glmer(cbind(alive, dead) ~ Gen10 + RH + High + Gen10xRH + Gen10xHigh

+ (1|Rep) + (1|Population) + (1|obs),

family="binomial", data=hd)

# Ensure fit1 and fit2 are the same model by performing a likelihood ratio test

anova(fit1,fit2)

# Post-hoc tests performed for each treatment contrast and used in assessing differences in survival between each selection experiment treatment

# Model contrasts for H0: High dose = Zero dose.

fit2c <- glmer(cbind(alive, dead) ~ Gen10 + RH + Gen10xRH

+ (1|Rep) + (1|Population) + (1|obs),

family="binomial", data=hd)

anova(fit2,fit2c)

# Model contrasts for H0: Random mortality = High dose.

fit2d <- glmer(cbind(alive, dead) ~ Gen10 + I(RH + High) + I(Gen10xRH + Gen10xHigh)

+ (1|Rep) + (1|Population) + (1|obs),

family="binomial", data=hd)

anova(fit2,fit2d)

# Model contrasts for H0: Random mortality = Zero dose.

fit2e <- glmer(cbind(alive, dead) ~ Gen10 + High + Gen10xHigh

+ (1|Rep) + (1|Population) + (1|obs),

family="binomial", data=hd)

anova(fit2,fit2e)
